# The role of adiponectin and AdipoR1/AKT signaling axis in mediating diabetic corneal epithelial wound healing and sensory nerve regeneration

**DOI:** 10.1186/s40662-025-00458-w

**Published:** 2025-10-27

**Authors:** Kelan Yuan, Wu Yaying, Chunyang Wang, Ning Wang, Yujie Mou, Ye Wang, Xiuming Jin, Shuo Yang

**Affiliations:** 1 Eye Center, The Second Affiliated Hospital, School of Medicine, Zhejiang Provincial Key Laboratory of Ophthalmology, Zhejiang Provincial Clinical Research Center for Eye Diseases, Zhejiang Provincial Engineering Institute On Eye Diseases, Zhejiang University, Hangzhou, 310009 China; 2 Core Laboratory, The Affiliated Qingdao Central Hospital of Qingdao University, Qingdao, 266042 Shandong China

**Keywords:** Adiponectin, AdipoR1/AKT signaling, Diabetic keratopathy, Corneal wound healing, Sensory nerve regeneration

## Abstract

**Purpose:**

Diabetic keratopathy, a common ocular complication of diabetes, is characterized predominantly by corneal epithelial damage and peripheral nerve injury. This study examined the role of adiponectin (ADPN) in regulating the repair of the diabetic corneal epithelium and accompanying nerve injuries.

**Methods:**

RNA sequencing was performed on total RNA isolated from corneal epithelium of streptozotocin (STZ)-induced type 1 diabetic mice and type 2 diabetic BKS.Cg-Dock7m +/+ Leprdb/Nju (db/db) mice to identify differentially regulated pathways and interactions. ADPN receptor expression was assessed. Recombinant ADPN, ADPN receptor 1/2 siRNA, and a phosphorylated AKT (p-AKT) inhibitor were then utilized in diabetic mice and in human corneal epithelial cells (HCECs) cultured under high-glucose conditions to evaluate corneal wound healing responses.

**Results:**

ADPN receptor expression and p-AKT levels were downregulated in corneas of diabetic mice and in HCECs exposed to high glucose. Treatment with recombinant ADPN accelerated repair of corneal epithelial and nerve damage in both type 1 and type 2 diabetic mice, enhanced HCEC proliferation and migration under high-glucose conditions and activated AKT signaling. ADPN treatment also reduced neutrophil infiltration and inflammatory factor expression during wound repair. These beneficial effects were abolished by ADPN receptor 1 knockdown or AKT inhibition.

**Conclusions:**

Our results demonstrate that ADPN promotes the corneal epithelium and nerve regeneration in diabetic mice via activation of the AdipoR1/AKT signaling axis and suppression of inflammatory responses. These findings identify ADPN as a promising therapeutic candidate for promoting corneal epithelial wound healing in diabetic conditions.

**Supplementary Information:**

The online version contains supplementary material available at 10.1186/s40662-025-00458-w.

## Background

Diabetic keratopathy (DK) has emerged as a major cause of vision-threatening complications in diabetic patients, characterized by corneal neurodegeneration, persistent epithelial defects, punctate keratitis, and neurotrophic ulcers [1, 2]. Diabetes-induced ocular surface barrier impairment severely compromises patient health and vision. These pathological alterations significantly disrupt ocular surface barrier function and corneal homeostasis, warranting increasing clinical attention.

The cornea exhibits high sensitivity to elevated glucose concentrations. Disruption of epithelial-neuro-immune cell (i.e., epineuroimmune) interactions is a primary driver of DK pathogenesis [1, 3]. Epithelial-derived cytokines and neurotrophic/axon-guidance factors modulate immune cell and corneal nerve function. Conversely, sensory nerve endings release neuropeptides that suppress inflammation and promote epithelial wound healing. Resident immune cells, including dendritic cells, provide neurotrophic factors and growth factors supporting neuronal and epithelial cells, respectively. Diabetes profoundly disrupts these interdependencies, suppressing epithelial cell proliferation, inducing sensory neuropathy, and reducing resident immune cell populations while increasing infiltrating immune cells, particularly neutrophils [4–6]. This imbalance exacerbates inflammation and contributes to neurotrophic keratopathy.

Adiponectin (ADPN), a bioactive hormone ubiquitously expressed in the body, ameliorates insulin resistance and inhibits diabetes progression. Previous studies indicate that serum ADPN levels can predict type 2 diabetes. Furthermore, ADPN has demonstrated anti-diabetic and anti-inflammatory effects in clinical trials [7–10]. Additional studies demonstrate that ADPN promotes proliferation and repair in diverse cell types within adipose tissue, bone, and skin [10–13]. Although the expression pattern of ADPN in diabetic corneal tissue remains unexplored, our preliminary data (Figure S1) revealed reduced ADPN expression in the corneal epithelium, serum, and trigeminal ganglion of db/db mice, suggesting that ADPN deficiency may contribute to DK pathogenesis. However, its role and mechanism within the epineuroimmune unit remain uncharacterized.

Adiponectin receptors, AdipoR1, AdipoR2, and T-cadherin (CDH13), mediate ADPN’s effects in various tissues [11, 14, 15]. Our preliminary RNA sequencing determined the expression profiles of corneal epithelium from control, type 1 diabetic (T1D), and type 2 diabetic (T2D) mice. Although T-cadherin (CDH13) is an established adiponectin receptor, our pre-study quantification revealed negligible expression in corneal tissues relative to AdipoR1/R2. Given this marginal abundance below functional thresholds for signaling dominance and alignment with established research paradigms prioritizing predominant receptors, we focused mechanistic investigations on AdipoR1 and AdipoR2 as the primary mediators. Protein–protein interaction (PPI) network analysis of differentially expressed genes (DEGs) revealed an association between AdipoR1/R2 and AKT signaling. We observed downregulation of AdipoR1 and R2 expression levels in the corneal epithelium of diabetic mice. However, the principal receptor mediating corneal healing during DK and the specific role of AKT signaling in this process remain elusive.

Upon binding to its receptor, ADPN initiates a cascade of tissue-specific signal transduction events, including the PI3K/AKT pathway [16]. AKT signaling regulates diverse cellular processes, such as apoptosis, cell proliferation, the cell cycle, protein synthesis, and glucose metabolism. This signaling pathway plays a critical role in the biological responses of corneal epithelial cells. Growth factors, including insulin-like growth factor 1 (IGF1) and insulin, activate AKT signaling through their respective receptors. This activation inhibits apoptosis and inflammation while simultaneously promoting the proliferation and migration of corneal epithelial cells, thereby accelerating epithelial wound healing [17, 18]. Conversely, diabetes-associated hyperglycemia directly inhibits AKT signaling. This inhibition occurs via increased generation of reactive oxygen species and endoplasmic reticulum stress within corneal epithelial cells, or through suppression of growth factor receptors, contributing to the pathogenesis of DK [19]. Several studies indicate that ADPN can promote proliferation in various cell types, such as B cells and human microvascular endothelial cells (HMEC-1), by modulating AKT signaling [20, 21]. However, it remains to be determined whether ADPN can enhance the function of the epineuroimmune unit via AKT signaling to alleviate DK.

Therefore, this study employed T1D and T2D murine models to assess the role of ADPN within the “epineuroimmune” functional unit and to investigate the involvement of AKT signaling. This investigation is critical for elucidating ADPN-induced functional recovery of the epineuroimmune unit in DK and to define the mechanism of the AdipoR1/AKT signaling axis in repairing the corneal epithelium and nerve damage in diabetic patients.

## Methods and materials

### Diabetic mouse models

This study employed both type 1 and type 2 diabetes mouse models. For the induction of type 1 diabetes, 6- to 8-week-old male C57BL/6 mice (Shanghai SLAC Laboratory Animal Co., Ltd.) received intraperitoneal injections of streptozotocin (STZ; Sigma-Aldrich, St. Louis, Missouri, USA) at a dosage of 0.13 mg/g body weight following a 14–16 h fasting period. From ≥ 12 week post-injection, blood glucose levels were measured to be 22.43 ± 2.93 mmol/L, confirming successful establishment of the type 1 diabetes model. For type 2 diabetes, 6- to 8-week-old male BKS.Cg-Dock7m +/+ Leprdb/Nju (db/db) mice and their non-diabetic littermates (db/+) were procured from Nanjing Cavens Laboratory Animal Co., Ltd. (Nanjing, China). The db/db mice exhibited characteristic hyperglycemia, with fasting blood glucose (FBG) levels reaching 30.8 ± 0.8 mmol/L after being fed for at least 12 weeks. For anesthesia, mice were intraperitoneally administered with a cocktail of ketamine (100 mg/kg) and xylazine (7 mg/kg). Upon completion of experimental procedures, euthanasia was humanely performed via intraperitoneal overdose injection of sodium pentobarbital (150 mg/kg).

All animal-related experimental procedures were conducted at the Laboratory Animal Center of the Second Affiliated Hospital, Zhejiang University School of Medicine. These experiments strictly adhered to institutional guidelines and ethical standards, as well as received official approval from the Institutional Animal Care and Use Committee (IACUC) of the Second Affiliated Hospital, Zhejiang University School of Medicine (No. 2018–038). The study protocol was rigorously compliant with the ARVO Statement for the Use of Animals in Ophthalmic and Vision Research established by the Association for Research in Vision and Ophthalmology.

### RNA sequencing

To characterize the gene expression profile of the mouse corneal epithelium, RNA sequencing (encompassing RNA extraction and library construction) was performed. Total RNA was extracted using TRIzol reagent following the manufacturer’s protocol. RNA quantity and purity were assessed using a Nanodrop 2000 spectrophotometer (Thermo Fisher Scientific, USA). RNA integrity was evaluated with an Agilent 2100 Bioanalyzer (Agilent Technologies, Santa Clara, CA, USA). Transcriptome libraries were constructed using the Vahts Universal V5 RNA-Seq Library Prep Kit according to the manufacturer’s instructions. These libraries were then sequenced on an Illumina NovaSeq 6000 platform to generate 150-bp paired-end reads. Transcriptome sequencing and subsequent bioinformatic analysis were performed by OE Biotech Co., Ltd. (Shanghai, China).

### Corneal epithelium wounds

Following anesthesia induction, a 2.5 mm diameter circular trephination was created in the central region of the mouse cornea using a corneal trephine. The corneal epithelium within this 2.5 mm circular area was then meticulously excised using an Algerbrush II corneal rust ring remover (Alger Co., Lago Vista, TX) under a surgical microscope.

### Drug and small interfering RNA (siRNA) treatment

Recombinant murine adiponectin (ADPN; PeproTech) was formulated as a 0.10% (w/v) eye drop solution with normal saline and administered topically four times daily (5 μL/dose) for 5 consecutive days prior to, and 2 or 7 days following, corneal epithelial wound induction. Mouse AdipoR1/R2-specific small interfering RNA (siRNA; 1.56 nmol/injection/eye, 5 μL volume; RiboBio Co., Ltd., China) or control siRNA was administered via subconjunctival injection 1 day prior to and on the day of corneal epithelial wound induction. For AKT signaling intervention, the AKT inhibitor triciribine was injected subconjunctivally 1 day prior to and on the day of wounding.

### Corneal sensitivity measurement

Corneal sensitivity in awake, non-anesthetized mice was assessed using a Cochet–Bonnet esthesiometer (Luneau Technology, France). Testing began with a filament length of 6 cm, which was progressively reduced in 0.5 cm increments until a response was elicited. The central corneal sensitivity threshold was defined as the maximum filament length at which mice exhibited a blink reflex. The measured filament length corresponding to this threshold was converted to actual pressure units, and the data were subjected to statistical analysis. For each mouse, corneal sensitivity was tested three times, and the mean value was calculated as the final sensitivity score.

### Human corneal epithelial cell (HCEC) culture and intervention

HCECs were purchased from the American Type Culture Collection (ATCC; Manassas, VA, USA) and maintained in Dulbecco’s Modified Eagle Medium/F12 (DMEM/F12; Corning, NY, USA) supplemented with 10% fetal bovine serum (FBS) and 1% penicillin-streptomycin (Gibco, USA). For experimental groups, the high-glucose (H-glu) group was treated with 35 mmol/L D-glucose, while mannitol was used as an osmotic control (H-op). After 24 h of incubation, a 200 μL sterile micropipette tip was used to scrape the cell monolayer to assess epithelial cell wound healing capacity. For recombinant human ADPN (rhADPN; 5 ng/mL; MCE, USA) treatment, the compound was added to H-glu-induced HCECs 2 h prior to cell layer scraping. Concurrently, during hyperglycemic stimulation, human AdipoR1/R2-specific siRNAs (RiboBio Co., Ltd., China) were transfected into HCECs using a customized siRNA transfection reagent system according to the manufacturer’s protocol. To inhibit AKT signaling, the AKT inhibitor triciribine (10 μM; Selleck) was administered 2 h before wound induction.

### Reverse transcription polymerase chain reaction (RT-PCR)

Mouse corneal epithelium was excised using an Algerbrush II corneal rust ring remover under a surgical microscope. Total RNA was extracted from both mouse corneal epithelium and HCECs using RNAiso Plus Reagent (TaKaRa, Shiga, Japan) following the manufacturer’s protocol. Complementary DNA (cDNA) synthesis was performed with HiScript II Q RT SuperMix (Vazyme, Shanghai, China) according to the provided instructions. Quantitative real-time PCR (qRT-PCR) was conducted using SYBR Green reagent (Vazyme) on a 7500 Fast Real-Time PCR System (Applied Biosystems, Waltham, MA, USA).

### Western blot analysis

Total protein was extracted from mouse corneal epithelial cells and HCECs. Cells were homogenized in ice-cold strong radio-immunoprecipitation assay (RIPA) lysis buffer (Beyotime Biotechnology, Jiangsu, China) containing protease and phosphatase inhibitors. After centrifugation at 12,000 × g for 15 min at 4 °C, supernatants containing total protein were collected. Protein concentrations were quantified using a bicinchoninic acid (BCA) assay kit (Sigma-Aldrich). Protein samples were separated by sodium dodecyl sulfate–polyacrylamide gel electrophoresis (SDS-PAGE) (GenScript, China) and subsequently transferred onto polyvinylidene fluoride (PVDF) membranes (Merck Millipore, Germany). Membranes were blocked with blocking buffer (Beyotime) at room temperature (24 °C) for 2 h, followed by overnight incubation with primary antibodies at 4 °C. The next day, membranes were incubated with corresponding secondary antibodies for 1.5 h at room temperature. The primary and secondary antibodies used in this study are listed in Table S1. Representative blots from three independent experiments are shown.

### Immunofluorescence staining

Immunofluorescence staining was performed on both frozen tissue sections and whole-mount mouse corneas. For frozen sections, eyeballs were dissected and embedded in optimal cutting temperature (OCT) compound (Sakura Finetek USA, Inc.), followed by sectioning into 8 μm thick cryosections. After fixation with 4% paraformaldehyde (PFA), sections were blocked for 1 h at room temperature (RT) with blocking buffer (composed of 0.5% Triton X-100, 3% BSA, 10% goat serum, and PBS in a ratio of 4.8 mL: 4 mL: 2.4 mL: 1 mL). Primary antibodies against AdipoR1 (Invitrogen, 1:100), AdipoR2 (Abcam, 1:100), Ki-67 (Abcam, 1:100), and p-AKT (Proteintech, 1:100) were then added, and sections were incubated overnight at 4 °C. Subsequently, corresponding secondary antibodies were applied for 1.5 h at RT.

For whole-mount corneal staining, eyeballs were harvested and fixed in 4% PFA at 4 °C for 1–2 h. The entire cornea was dissected from the eyeball, blocked with blocking buffer for 2 h at RT then incubated overnight at 4 °C with either neuron-specific β-III tubulin conjugated to NL557 (R&D Systems, 1:50) or neutrophil-specific Ly6G-FITC-conjugated antibody (Sigma-Aldrich, 1:50). Neurite outgrowth and neutrophil distribution were analyzed and quantified using NeuronJ, an ImageJ plug-in for nerve tracing.

For HCECs, cells were cultured on glass slides, fixed with 4% PFA for 15 min, and then blocked with blocking buffer for 1 h at RT. Slides were then incubated overnight at 4 °C with primary antibodies against AdipoR1 (Invitrogen, 1:100), AdipoR2 (Thermo Fisher, 1:100), Ki-67 (Abcam, 1:100), and p-AKT (Proteintech, 1:100), followed by 1.5 h incubation with their corresponding secondary antibodies at RT.

### Statistical analysis

Data are presented as mean ± standard deviation (SD). Statistical analyses were conducted using GraphPad Prism 7.0 software (GraphPad Software Inc., San Diego, CA, USA). For intergroup comparisons, one-way analysis of variance (ANOVA) followed by Dunnett’s multiple comparisons test (for multiple groups) or unpaired two-tailed Student’s t-test (for two groups) was applied. Statistical significance was defined as *P < 0.05, **P < 0.01, and ***P < 0.001.

## Results

### ADPN/AdipoR1/R2 expression in the diabetic mouse cornea and H-glu-cultured HCECs

**Fig. 1:**
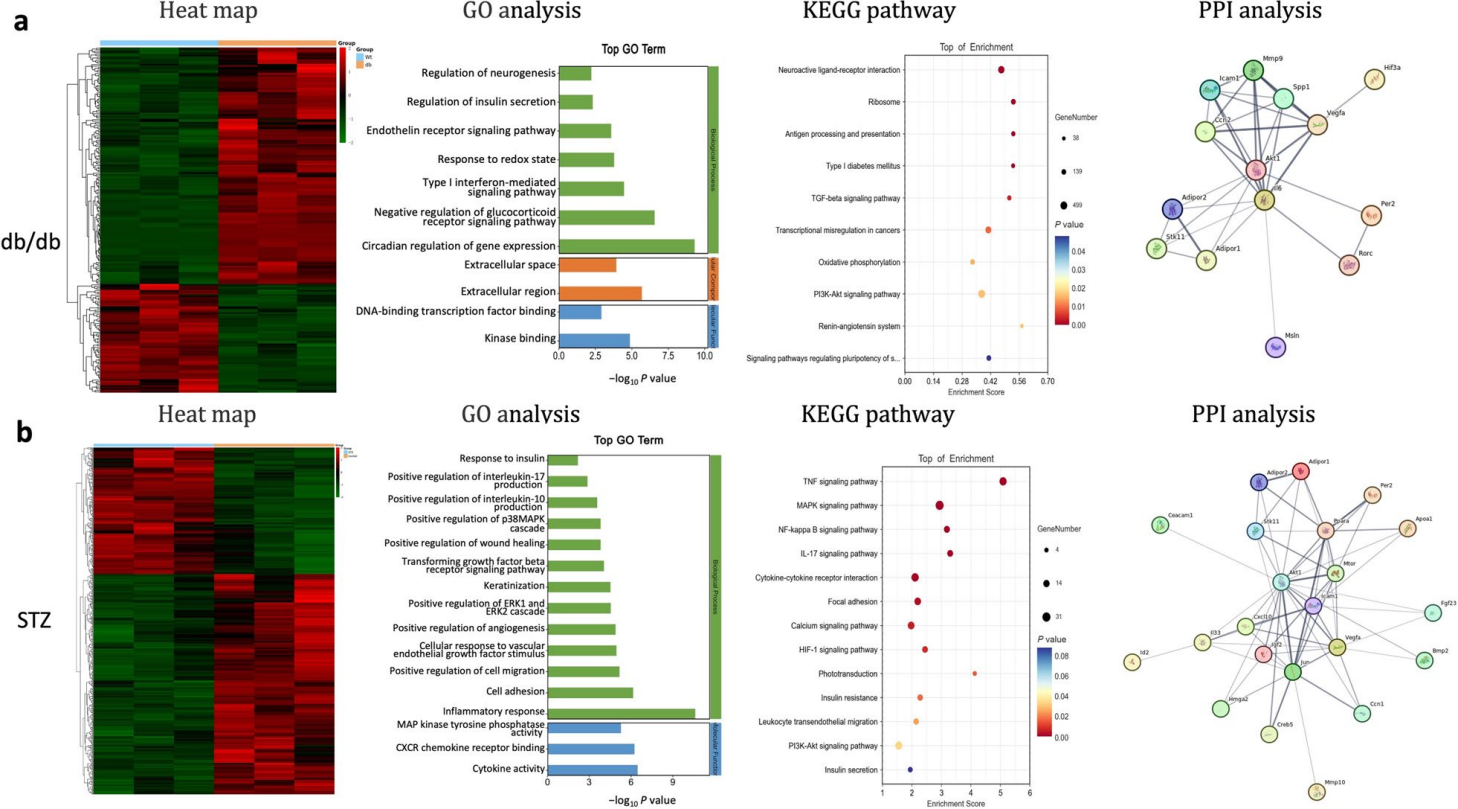
RNA sequencing analysis of corneal epithelial gene expression profiles in control, type 1 diabetic (T1D), and type 2 diabetic (T2D) mice. RNA sequencing was performed on corneal epithelial samples from T1D (streptozotocin-induced), T2D (db/db), and age-matched control mice prior to corneal epithelial injury (n = 3 per group). **a** Heatmap, Gene Ontology (GO) enrichment, Kyoto Encyclopedia of Genes and Genomes (KEGG) pathway, and protein–protein interaction (PPI) network analyses of differentially expressed genes (DEGs) in db/db mice. **b** Corresponding analyses for STZ-induced T1D mice

First, we successfully established and validated two widely used murine models of diabetes: STZ-induced T1D mice and BKS.Cg-Dock7m +/+ Leprdb/Nju (db/db) T2D mice (Figures S2, S3). To characterize diabetes-specific corneal molecular signatures prior to injury, we performed RNA sequencing (RNA-seq) on uninjured corneal tissues from control, T1D, and T2D mice (Fig. 1). This analysis revealed distinct transcriptional profiles, providing critical insights for subsequent investigations. Transcriptomic profiling under basal conditions identified diabetes-specific molecular signatures. Gene Ontology (GO) and Kyoto Encyclopedia of Genes and Genomes (KEGG) enrichment analyses demonstrated that DEGs were primarily associated with wound healing, neurogenesis, inflammatory responses, insulin signaling, and the PI3K-AKT and pathways (Fig. 1a, b). PPI network analysis further identified AdipoR1 and AdipoR2 as key interactors within the AKT signaling cascade (Fig. 1a, b).

**Fig. 2:**
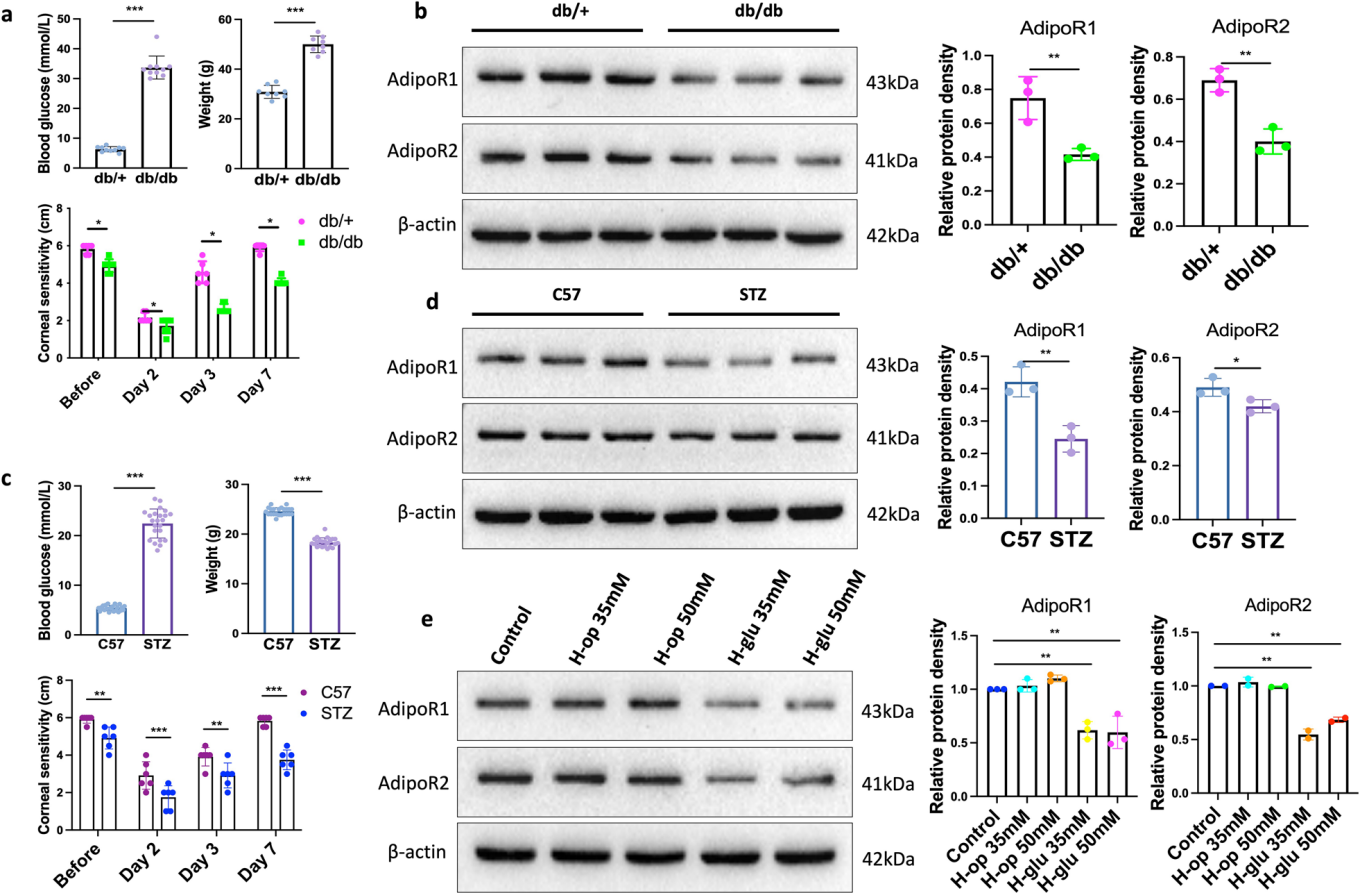
AdipoR1/R2 expression in diabetic mice and high-glucose-exposed human corneal epithelial cells (HCECs). **a**, **c** Temporal changes in blood glucose levels, body weight, and corneal sensitivity in db/db (**a**) and STZ-induced T1D (**c**) mice compared with controls. **b**, **d** AdipoR1/R2 protein expression in T2D (**b**, n = 3) and T1D (**d**, n = 3) mouse corneas. **e** AdipoR1/R2 protein levels in HCECs exposed to high glucose (H-glu, n = 3). Representative blots from three independent experiments. Data are presented as mean ± SD. Statistical analyses: one-way ANOVA with Dunnett’s test (multiple groups) or unpaired t-test (two groups). T1D, type 1 diabetic; T2D, type 2 diabetic. *P < 0.05, **P < 0.01, ***P < 0.001

Next, we assessed body weight and blood glucose levels in both diabetic models (T1D and T2D) alongside age-matched controls (Fig. 2a, c). The db/db mice exhibited severe hyperglycemia (30.8 ± 0.8 mmol/L vs. control: 6.32 ± 0.27 mmol/L, *P* < 0.001) and increased body weight (50.0 ± 3.33 g vs. control: 31.70 ± 2.58 g, *P* < 0.001) compared with age-matched controls. In contrast, STZ-treated T1D mice displayed reduced body weight (18.32 ± 0.18 g vs. control: 24.55 ± 0.15 g, *P* < 0.001) and hyperglycemia (22.43 ± 2.93 mmol/L vs. control: 5.37 ± 0.11 mmol/L, *P* < 0.001). Corneal sensitivity was quantitatively evaluated at baseline (pre-injury) and at 2, 3, and 7 days post-injury using standardized protocols (Fig. 2a, c). Results indicated that diabetic mice exhibited reduced corneal sensitivity both before and after injury compared with controls.

We then validated the protein expression of AdipoR1 and AdipoR2 in murine and cellular models of diabetes. AdipoR1 expression was significantly downregulated in the corneal epithelium of both T1D and T2D mice (Fig. 2b, d). To investigate the effects of hyperglycemic stress on AdipoR1/R2 expression, we used isotonic sorbitol (H-op) as an osmotic control. No significant changes in AdipoR1/R2 protein expression were observed between HCECs exposed to H-op and normal controls, whereas a marked reduction was detected in H-glu-treated HCECs (Fig. 2e). Immunofluorescence analysis revealed distinct spatial distribution patterns of AdipoR1 and AdipoR2 in normal murine corneas (Figure S4). Notably, AdipoR1 exhibited a pan-corneal distribution, with immunoreactivity detected across all corneal layers, including the epithelium, stroma, and endothelium. Similarly, robust immunostaining for both AdipoR1 and AdipoR2 was consistently observed in HCECs under physiological conditions (Figure S5).

### ADPN significantly accelerated repair of corneal epithelium injuries and promoted nerve regeneration while suppressing neutrophil infiltration

**Fig. 3:**
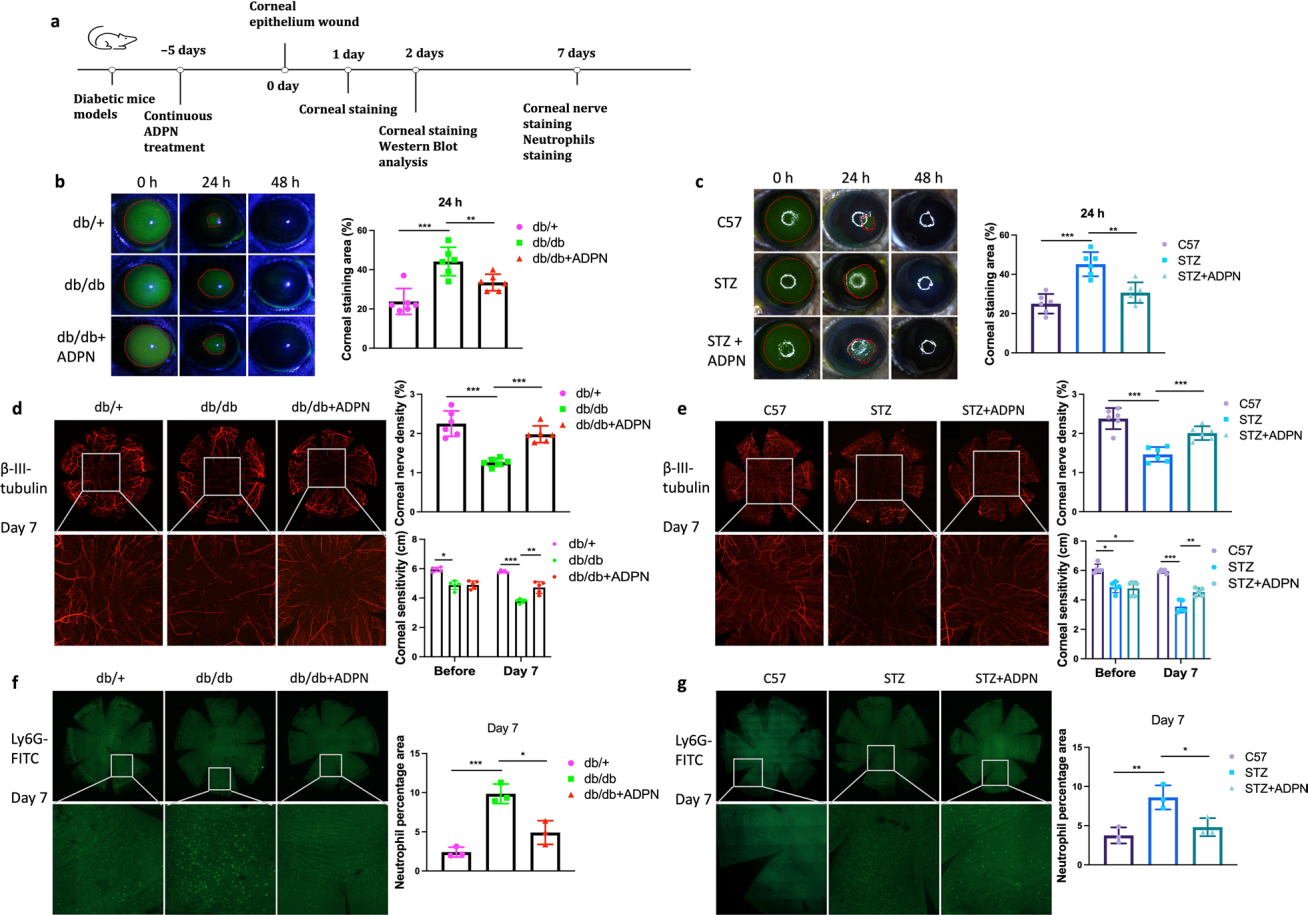
Adiponectin (ADPN) enhances corneal epithelial repair, nerve regeneration, and reduces neutrophil infiltration in diabetic mice.** a** Experimental timeline. **b**, **c** Corneal epithelial wound closure assessed by fluorescein sodium staining in ADPN-treated T2D (**b**, n = 6) and T1D (**c**, n = 6) mice. **d**, **e** Corneal nerve regeneration (β-III tubulin immunofluorescence, n = 6) and corneal sensitivity (Cochet-Bonnet esthesiometer, n = 5) in ADPN-treated T2D (**d**) and T1D (**e**) mice. **f**, **g** Neutrophil accumulation (Ly6G immunofluorescence, n = 3) in ADPN-treated T2D (**f**) and T1D (**g**) mice. Data are presented as mean ± SD. Statistical analyses: one-way ANOVA with Dunnett’s test (multiple groups) or unpaired t-test (two groups). T1D, type 1 diabetic; T2D, type 2 diabetic; STZ, streptozotocin. *P < 0.05, **P < 0.01, ***P < 0.001

To investigate the role of ADPN in the “epineuroimmune” axis during corneal repair, we evaluated its effects on epithelial regeneration, nerve innervation, and neutrophil dynamics in diabetic mice (Fig. 3a). Corneal epithelial repair was assessed via fluorescein sodium staining at 0, 24, and 48 h post-scratching. In both T2D and T1D mice, the epithelial defect area at 24 h was significantly larger than in vehicle-treated controls (Fig. 3b, c). Notably, ADPN administration markedly accelerated epithelial wound closure in diabetic corneas (Fig. 3b, c).

Nerve regeneration was evaluated using whole-mount immunofluorescence staining for neuron-specific β-III tubulin. At 1 week post-injury, ADPN-treated db/db and STZ-induced diabetic mice exhibited significantly increased corneal nerve density compared to vehicle-treated counterparts (Fig. 3d, e). Consistent with this structural improvement, corneal sensitivity, measured via the Cochet-Bonnet esthesiometer, was significantly enhanced in ADPN-treated diabetic mice versus vehicle controls, correlating with the observed nerve density differences (Fig. 3d, e).

As key components of the innate immune response, neutrophils were analyzed using Ly6G-specific immunofluorescence. Under normal conditions (db/+ and C57 control groups), neutrophils were scarce in the cornea. However, hyperglycemic environments (type 1 and type 2 diabetes) induced robust neutrophil recruitment at the injury site. ADPN treatment significantly reduced neutrophil accumulation at the wound area (Fig. 3f, g).

**Fig. 4:**
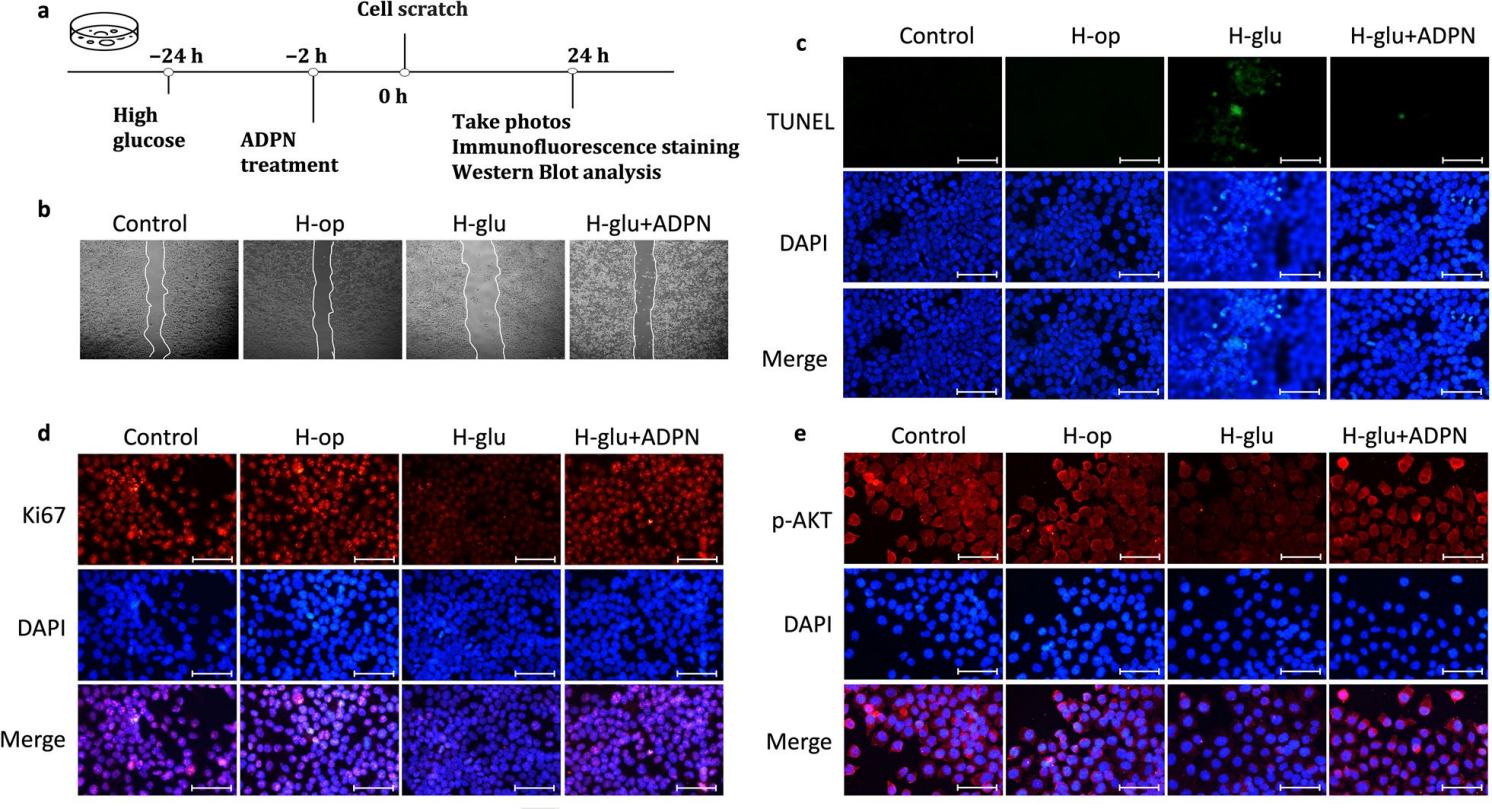
Adiponectin (ADPN) promotes human corneal epithelial cell (HCEC) migration and proliferation under high glucose conditions.** a** Schematic of in vitro experiments. **b** Cell migration assessed by scratch wound assay (n = 3). (**c**) Apoptotic cells (TUNEL staining, green, n = 3), (**d**) Ki-67 expression (immunofluorescence, n = 3), and (**e**) p-AKT expression (immunofluorescence, n = 3) after ADPN treatment in HCECs. Representative images are from three independent experiments. Scale bars represent 75 μm. H-op, high-osmotic control; H-glu, high-glucose

In vitro studies further validated ADPN’s effects in H-glu-stressed HCECs, with isotonic mannitol (H-op) serving as an osmotic control (Fig. 4a). Scratch wound assays revealed no significant difference in cell migration between H-op-treated and normal HCECs. In contrast, H-glu treatment markedly impaired migration, which was rescued by ADPN supplementation (Fig. 4b). Terminal deoxynucleotidyl transferase dUTP nick end labeling (TUNEL) staining demonstrated that ADPN attenuated H-glu-induced HCEC apoptosis (Fig. 4c). Additionally, H-glu suppressed the proliferative marker Ki-67, whereas ADPN treatment preserved Ki-67 expression (Fig. 4d).

### ADPN promotes mouse corneal epithelial repair primarily through AdipoR1

**Fig. 5:**
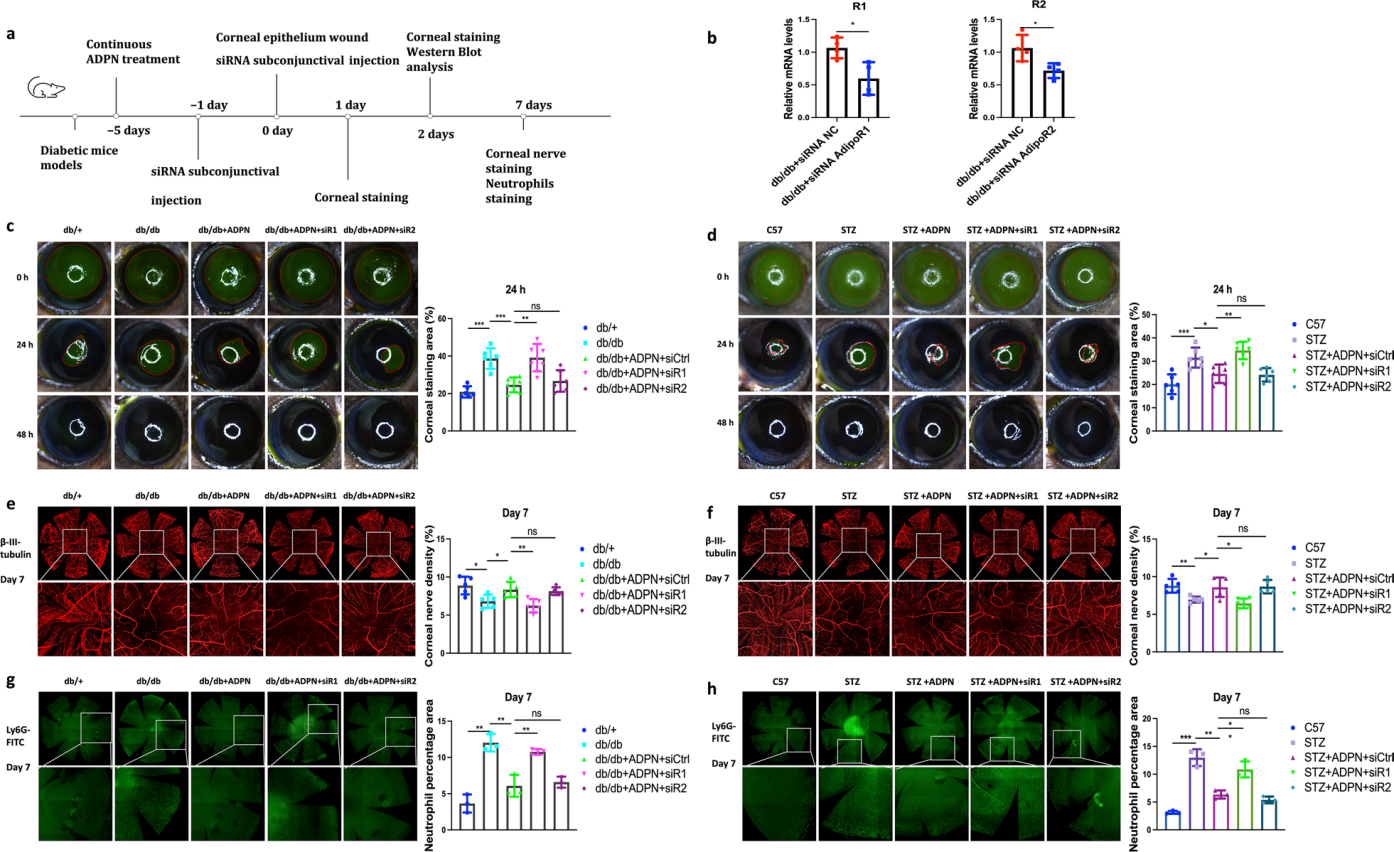
Adiponectin (ADPN) promotes corneal repair via AdipoR1 rather than AdipoR2 in diabetic mice.** a** Experimental design for siRNA-mediated AdipoR1/R2 knockdown. **b** AdipoR1/R2 mRNA levels in T2D mice post-siRNA (n = 4). **c**, **d** Corneal epithelial wound closure in T2D (**c**, n ≥ 6) and T1D (**d** n ≥ 6) mice post-siRNA. **e**, **f** β-III tubulin immunofluorescence (nerve density) in T2D (**e**, n = 5) and T1D (**f**, n = 5) mice. **g**, **h** Ly6G immunofluorescence (neutrophil infiltration) in T2D (**g**, n = 3) and T1D (**h**, n = 3) mice. Data are presented as mean ± SD. Statistical analyses: one-way ANOVA with Dunnett’s test (multiple groups) or unpaired t-test (two groups). T1D, type 1 diabetic; T2D, type 2 diabetic; siCtrl, control siRNA; siR1, AdipoR1 siRNA; siR2, AdipoR2 siRNA; STZ, streptozotocin. *P < 0.05, **P < 0.01, ***P < 0.001

**Fig. 6:**
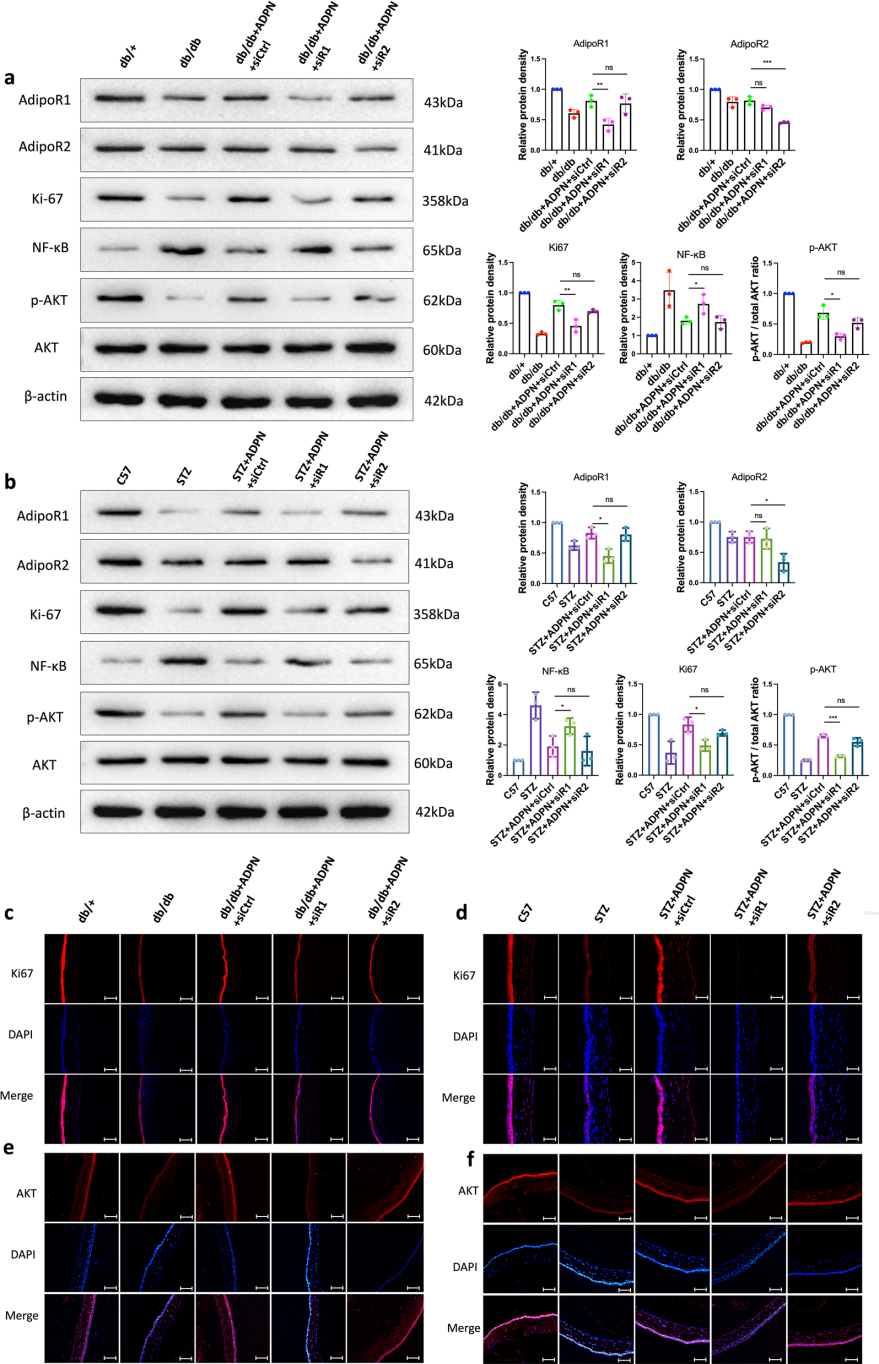
AdipoR1/R2 knockdown modulates key signaling molecules in diabetic mouse corneas.** a, b** Western blot analysis of AdipoR1, AdipoR2, Ki-67, NF-κB, p-AKT, and AKT in T2D (**a**, n = 3) and T1D (**b**, n = 3) mouse corneas post-siRNA. **c**, **d** Ki-67 immunofluorescence in T2D (**c**, n = 3) and T1D (**d**, n = 3) mice. **e**, **f** p-AKT immunofluorescence in T2D (**e**, n = 3) and T1D (**f**, n = 3) mice. Representative blots/images from three independent experiments. Scale bars represent 100 μm. Data are presented as mean ± SD. Statistical analyses: one-way ANOVA with Dunnett’s test (multiple groups) or unpaired t-test (two groups). NF-κB, nuclear factor kappa-light-chain-enhancer of activated B cells; p-AKT, phosphorylated AKT; AKT, protein kinase B; T1D, type 1 diabetic; T2D, type 2 diabetic; siCtrl, control siRNA; siR1, AdipoR1 siRNA; siR2, AdipoR2 siRNA; STZ, streptozotocin; ADPN, adiponectin. *P < 0.05, **P < 0.01, ***P < 0.001

To determine whether ADPN exerts its pro-repair effects through AdipoR1 or AdipoR2, we performed AdipoR1- or AdipoR2-specific siRNA-mediated knockdown (Fig. 5a). Successful silencing of AdipoR1/R2 in diabetic mice was confirmed by immunofluorescence staining (Figure S6), quantitative PCR (qPCR; Fig. 5b), and Western blotting (Fig. 6a, b). In both type 2 (Fig. 5c) and type 1 diabetes (Fig. 5d) models, knockdown of AdipoR1 nearly abrogated the pro-repair effect of ADPN on corneal epithelial injury, whereas AdipoR2 silencing had no such impact, indicating that ADPN promotes corneal epithelial repair specifically through AdipoR1.

To further investigate the role of AdipoR1 in ADPN-mediated nerve repair and neutrophil regulation, we assessed β-III tubulin (nerve marker) and Ly6G (neutrophil marker) using whole-mount immunofluorescence. In T2D mice, ADPN treatment significantly increased corneal nerve fiber density and improved corneal sensitivity compared to untreated controls. These effects were reversed by AdipoR1 knockdown but remained unaffected by AdipoR2 silencing (Fig. 5e). Consistent results were observed in T1D mice (Fig. 5f). For neutrophil recruitment, ADPN treatment reduced neutrophil accumulation in both diabetic models; however, AdipoR1 knockdown restored neutrophil numbers at the injury site (Fig. 5g, h).

Western blot analysis revealed that AdipoR1 expression was significantly downregulated in the corneal epithelium of both diabetic models, and this reduction was partially rescued by exogenous ADPN (Fig. 6a, b). Additionally, ADPN alleviated hyperglycemia-induced downregulation of Ki-67 (a cell proliferative marker), an effect that was partially reversed by AdipoR1 knockdown but not AdipoR2 silencing (Fig. 6a, b). Similarly, ADPN suppressed the marked upregulation of NF-κB (a pro-inflammatory transcription factor) in the diabetic corneal epithelium, whereas AdipoR1 knockdown restored NF-κB expression to baseline levels (Fig. 6a, b).

Immunofluorescent analysis of Ki67 distribution further confirmed these findings: ADPN treatment increased Ki67-positive staining in corneal epithelial cells, and this effect was attenuated by AdipoR1 siRNA but not AdipoR2 siRNA (Fig. 6c, d).

### ADPN promotes cell migration through AdipoR1 in an HCEC hyperglycemia model

**Fig. 7:**
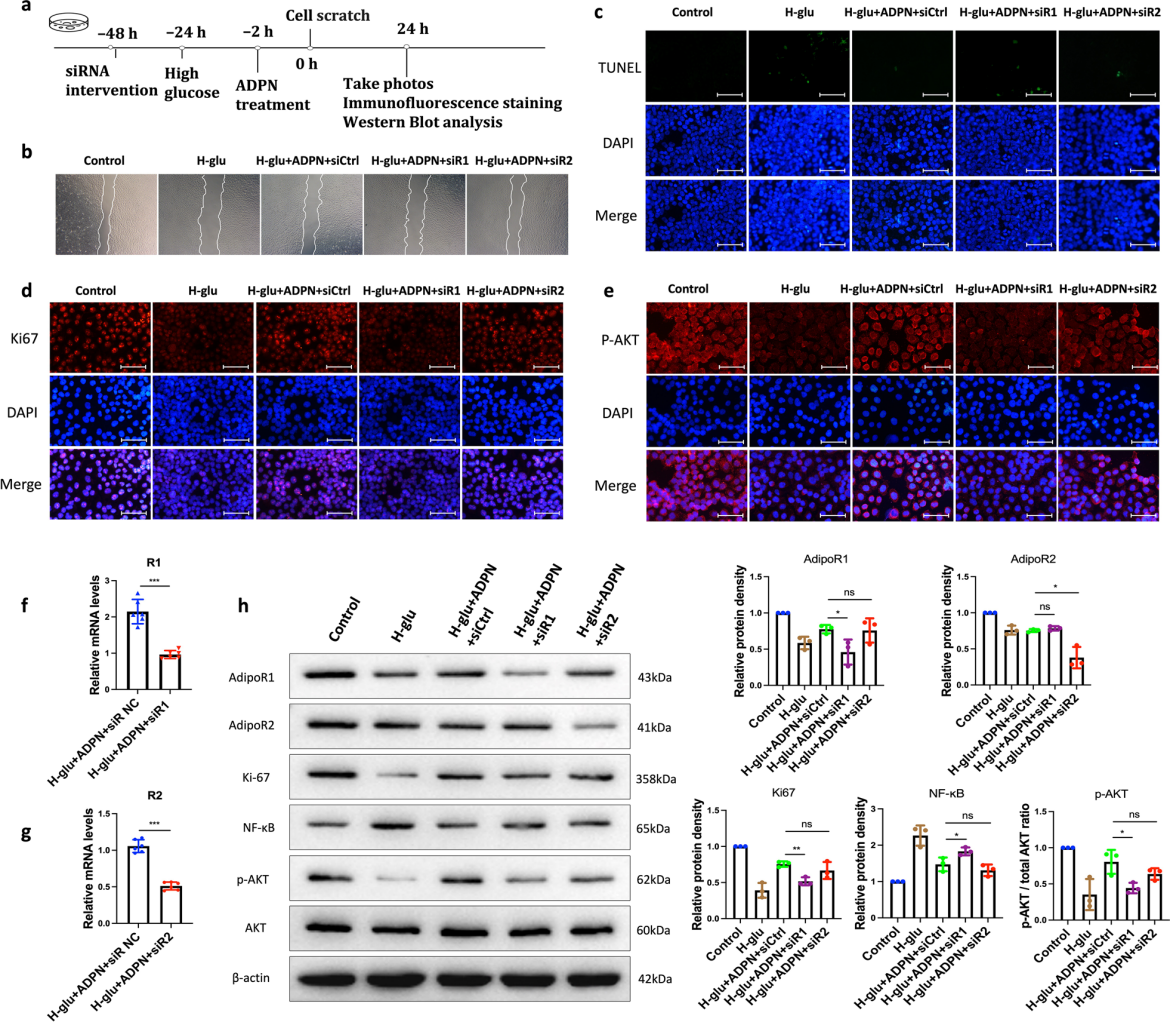
Adiponectin (ADPN) promotes human corneal epithelial cell (HCEC) migration via AdipoR1 rather than AdipoR2 under high glucose conditions. **a** Schematic of in vitro experiments with siRNA-mediated AdipoR1/R2 knockdown in HCECs. **b** Cell migration (scratch wound assay, n = 3). **c** Apoptotic cells (TUNEL staining, green, n = 3). **d** Ki-67 expression (n = 3). **e** p-AKT expression (n = 3). **f**, **g** siRNA knockdown efficiency (qPCR, n = 6). **h** Western blot of AdipoR1, AdipoR2, Ki-67, NF-κB, p-AKT, and AKT (n = 3). Representative images/blots from three independent cell experiments. Scale bars represent 75 μm. Data are presented as mean ± SD. Statistical analyses: one-way ANOVA with Dunnett’s test (multiple groups) or unpaired t-test (two groups). p-AKT, phosphorylated AKT; siRNA, small interfering RNA; qPCR, quantitative polymerase chain reaction; AKT, protein kinase B; H-glu, high-glucose; siCtrl, control siRNA; siR1, AdipoR1 siRNA; siR2, AdipoR2 siRNA; DAPI, 4′,6-Diamidino-2-Phenylindole. *P < 0.05, **P < 0.01, ***P < 0.001

To determine whether AdipoR1 or AdipoR2 mediates ADPN’s effects in H-glu stress, we transfected HCECs cultured under H-glu conditions with AdipoR1- or AdipoR2-specific siRNAs (Fig. 7a). siRNA interference efficiency was validated via qPCR (Fig. 7f, g) and Western blotting (Fig. 7h). Hyperglycemic stress delayed wound healing, which was rescued by ADPN treatment. Notably, AdipoR1-targeting siRNA significantly reduced the wound healing rate, whereas AdipoR2 siRNA transfection had no observable effect (Fig. 7b).

ADPN treatment attenuated TUNEL-positive staining (apoptosis marker) in HCECs; this protective effect was abrogated by AdipoR1 siRNA but not AdipoR2 siRNA (Fig. 7c). Additionally, ADPN increased the proportion of Ki-67^+^ cells and Ki-67 protein expression, whereas AdipoR1 siRNA transfection reversed this upregulation (Fig. 7d, h). Furthermore, ADPN suppressed the marked upregulation of NF-κB (a pro-inflammatory transcription factor) induced by H-glu stress, an effect that was restored by AdipoR1 siRNA (Fig. 7h).

### ADPN-AdipoR1 signaling may regulate corneal injury repair in diabetic mice via the AKT pathway

Our data demonstrated a significant downregulation of phosphorylated AKT (p-AKT) expression in the corneal epithelium of diabetic mice (Fig. 6a, b, e, f) and in H-glu-stressed HCECs (Figs. 4e, 7e and h). Notably, ADPN supplementation potently restored p-AKT levels, an effect that was effectively abolished by AdipoR1 knockdown. Immunofluorescence imaging of p-AKT (rather than total AKT) provided spatial insights into pathway activation within corneal epithelial cells (Figs. 4e, 6e, f, and 7e). Western blot analyses (Figs. 6a, b, and 7h) further confirmed that ADPN specifically enhances AKT phosphorylation without altering total AKT expression.

**Fig. 8:**
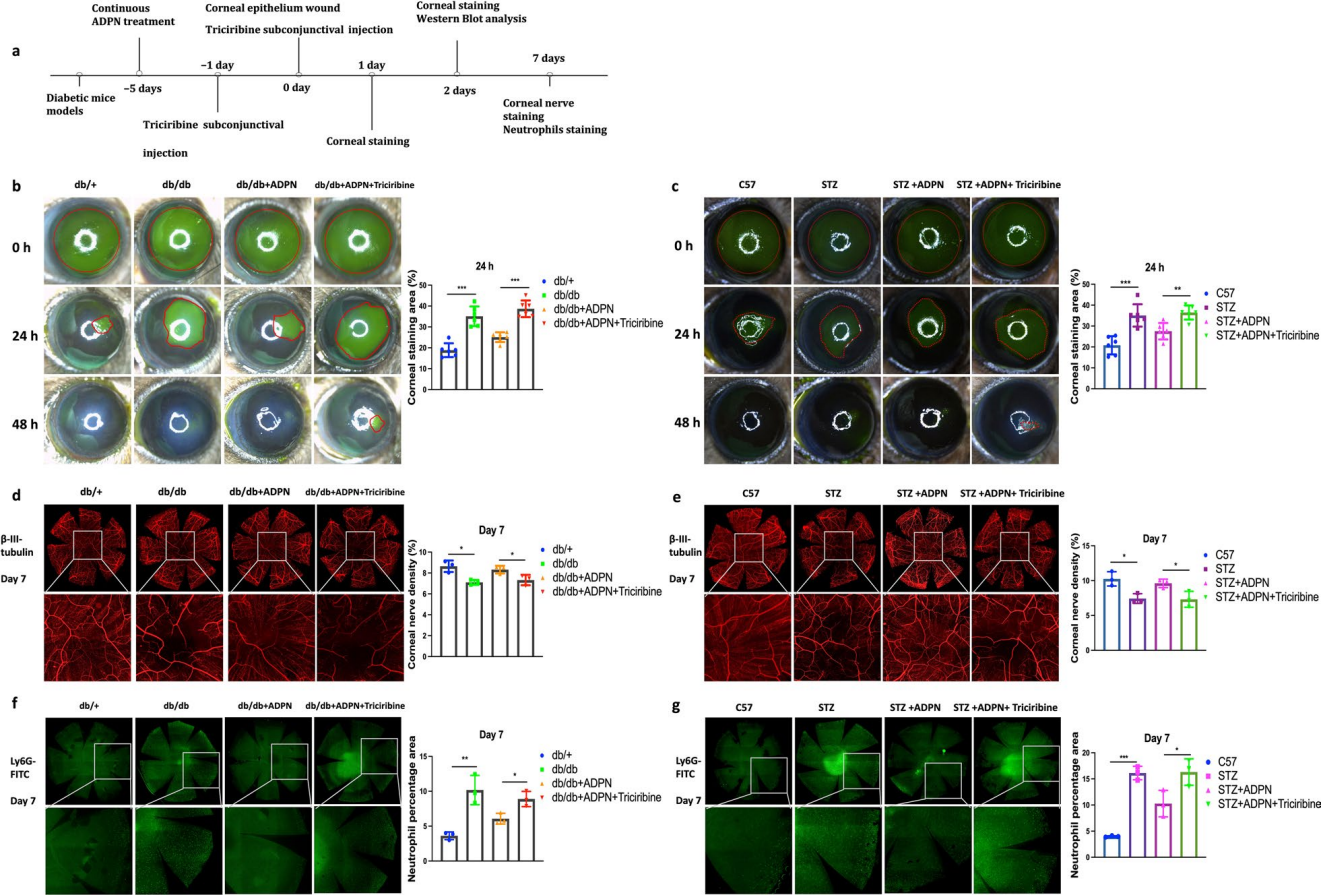
Adiponectin (ADPN)-AdipoR1 signaling regulates corneal repair via AKT activation in diabetic mice.** a** Experimental timeline for AKT inhibitor (triciribine) administration. **b**, **c** Corneal epithelial wound closure in T2D (**b**, n = 6) and T1D (**c**, n = 6) mice post-AKT inhibition. **d**, **e** β-III tubulin immunofluorescence (nerve density) in T2D (**d**, n = 3) and T1D (**e**, n = 3) mice. **f**, **g** Ly6G immunofluorescence (neutrophil infiltration) in T2D (**f**, n = 3) and T1D (**g**, n = 3) mice. Data are presented as mean ± SD. Statistical analyses: one-way ANOVA with Dunnett’s test (multiple groups) or unpaired t-test (two groups). T1D, type 1 diabetic; T2D, type 2 diabetic; STZ, streptozotocin. *P < 0.05, **P < 0.01, ***P < 0.001

To further investigate the involvement of this signaling axis in ADPN-induced corneal epithelial repair, we subconjunctivally administered the AKT inhibitor triciribine to diabetic mice (Fig. 8a). Fluorescein sodium staining revealed that ADPN treatment promoted corneal epithelial wound closure; however, this reparative effect was significantly blunted by AKT inhibition in both T2D (Fig. 8b) and T1D mice (Fig. 8c).

Immunofluorescence staining of β-III tubulin (a neuronal marker) demonstrated that ADPN treatment increased corneal nerve fiber density in db/db mice, which was reversed by AKT inhibition (Fig. 8d). Consistent results were observed in STZ-induced T1D mice (Fig. 8e). For neutrophil dynamics, ADPN treatment markedly reduced neutrophil recruitment in db/db (Fig. 8f) and STZ-treated mice (Fig. 8g); this anti-inflammatory effect was abrogated by AKT inhibition, leading to a significant rebound in neutrophil infiltration.

**Fig. 9:**
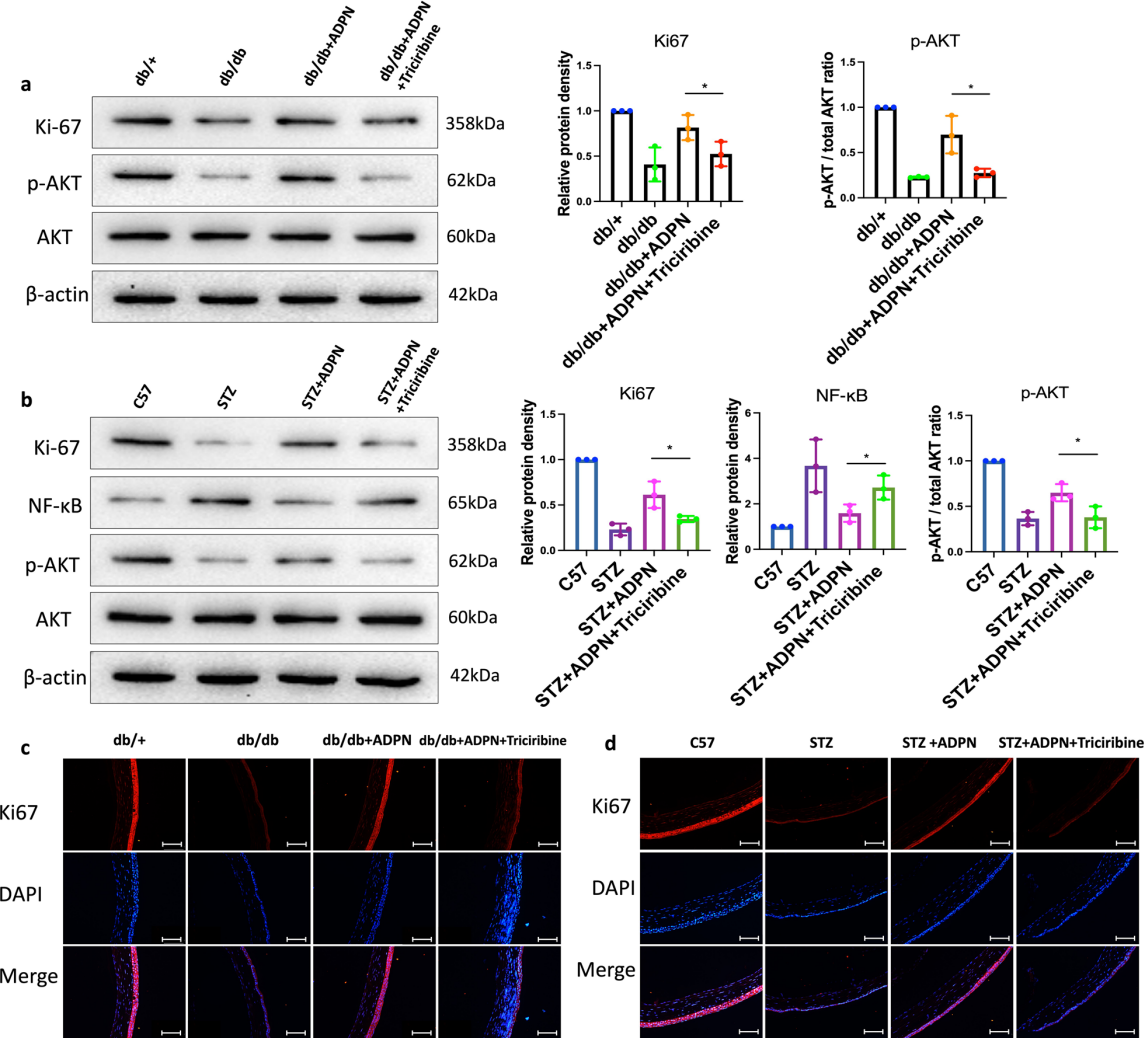
AKT inhibition modulates key signaling molecules in diabetic mouse corneas. **a**, **b** Western blot analysis of Ki-67, p-AKT and AKT in T2D (**a**) and T1D (**b**) mouse corneas post-AKT inhibition. **c**, **d** Ki-67 immunofluorescence in T2D (**c**, n = 3) and T1D (**d**, n = 3) mice. Representative blots/images from three independent experiments. Scale bars represent 100 μm. Data are presented as mean ± SD. Statistical analyses: one-way ANOVA with Dunnett’s test (multiple groups) or unpaired t-test (two groups). T1D, type 1 diabetic; T2D, type 2 diabetic; p-AKT, phosphorylated AKT; AKT, protein kinase B; STZ, streptozotocin; ADPN, adiponectin. *P < 0.05, **P < 0.01, ***P < 0.001

Western blot analysis further demonstrated that hyperglycemia-induced downregulation of Ki-67 (a proliferation marker) and p-AKT was partially rescued by ADPN. Notably, this restorative effect of ADPN on both Ki-67 and p-AKT levels was partially attenuated by AKT inhibition (Fig. 9a, b). Immunofluorescent staining of Ki-67 confirmed this trend, with ADPN-treated diabetic corneas exhibiting increased Ki-67^+^ cells that was reversed by AKT inhibition (Fig. 9c, d).

### ADPN-AdipoR1 signaling may regulate HCEC migration under hyperglycemia via p-AKT activation

**Fig. 10:**
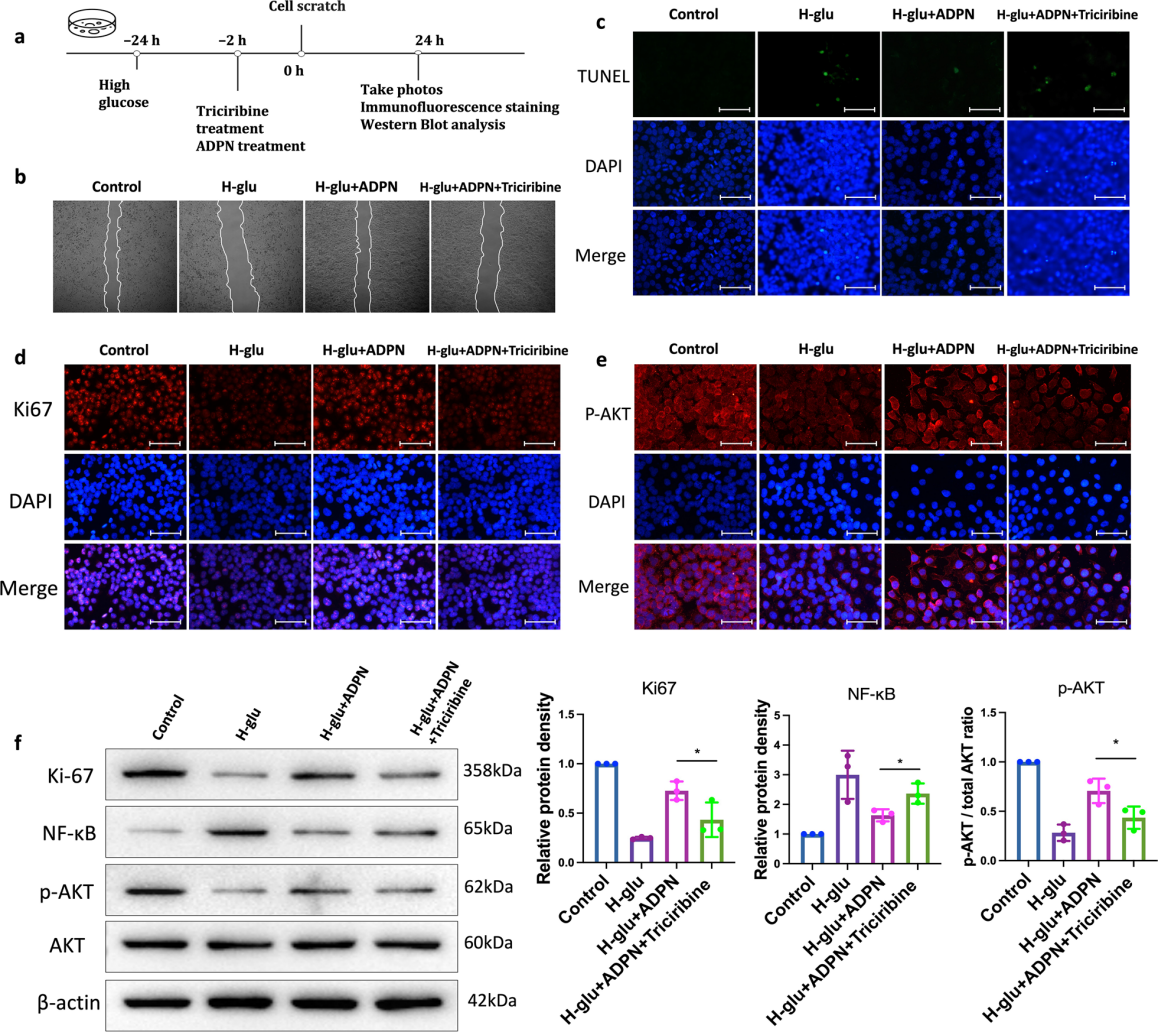
Adiponectin (ADPN)-AdipoR1 signaling promotes human corneal epithelial cell (HCEC) migration via AKT activation under high glucose conditions.** a** Schematic of in vitro experiments with AKT inhibitor (triciribine) in HCECs. **b** Cell migration (scratch wound assay, n = 3). **c** Apoptotic cells (TUNEL staining, green, n = 3). **d** Ki-67 expression (n = 3). **e** p-AKT expression (n = 3). **f** Western blot of Ki-67, NF-κB, p-AKT, and AKT (n = 3). Representative images/blots from three independent experiments. Scale bars represent 75 μm. Data are presented as mean ± SD. Statistical analyses: one-way ANOVA with Dunnett’s test (multiple groups) or unpaired t-test (two groups). AKT, protein kinase B; p-AKT, phosphorylated AKT; NF-κB, nuclear factor kappa-light-chain-enhancer of activated B cells; H-glu, high-glucose; DAPI, 4′,6-Diamidino-2-Phenylindole. *P < 0.05, **P < 0.01, ***P < 0.001

To determine whether AKT inhibition suppresses ADPN-induced activation of the AdipoR1/AKT signaling axis in a hyperglycemic cellular model, we conducted further investigations (Fig. 10a). The pro-migratory effect of ADPN on HCECs was significantly blunted by AKT inhibition (Fig. 10b). Additionally, ADPN treatment reduced TUNEL^+^ staining (apoptotic marker) in HCECs, whereas this protective effect was attenuated by AKT inhibition (Fig. 10c). Ki-67 immunostaining revealed that ADPN increased the proportion of proliferating HCECs, an effect that was reversed upon AKT inhibition (Fig. 10d).

Western blot analysis confirmed these observations: ADPN treatment upregulated Ki-67 expression in H-glu-stressed HCECs, which was substantially diminished by AKT inhibition (Fig. 10f). Similarly, hyperglycemia-induced NF-κB upregulation was suppressed by ADPN, whereas AKT inhibition restored NF-κB expression to baseline levels (Fig. 10f).

## Discussion

The intact corneal epithelium serves as a critical barrier against ocular infections; thus, rapid restoration of epithelial integrity is essential for visual recovery. While corneal epithelial defects in normoglycemic individuals heal efficiently, diabetic patients often exhibit delayed wound healing and reduced corneal sensitivity [22, 23]. Despite the clinical significance of DK, its pathogenesis remains poorly understood, and effective therapeutic strategies are limited. This study demonstrates that ADPN significantly promotes corneal epithelial wound healing, stimulates corneal nerve repair, and reduces neutrophil accumulation in both type 1 and type 2 diabetes models. Mechanistically, silencing AdipoR1 (but not AdipoR2) abrogates ADPN-mediated repair, impairs nerve regeneration, and exacerbates neutrophil infiltration. Furthermore, ADPN-AdipoR1 signaling likely regulates corneal injury repair in diabetes through activation of the AKT pathway.

A growing body of evidence underscores the importance of epithelial cell-immune cell-sensory neuron crosstalk in maintaining corneal homeostasis, a process disrupted in diabetes [5, 24]. Diabetes mellitus is categorized into type 1, type 2, and other subtypes [25, 26]. Here, we established two murine diabetes models: STZ-induced type 1 diabetes and leptin receptor knockout (db/db)-induced type 2 diabetes, along with a H-glu-stressed HCEC model. Both diabetic mouse models exhibited reduced corneal nerve fiber density and length, consistent with impaired sensory innervation. Corneal sensory nerves release neuropeptides that regulate epithelial and immune cell function [27–29]. Under normal conditions, neutrophils undergo apoptosis after contributing to wound resolution [30, 31]; however, hyperglycemia disrupts neutrophil metabolism and function [32]. Our findings align with these observations: H-glu environments promoted neutrophil infiltration, exacerbating inflammation, cell death, and delayed re-epithelialization and nerve regeneration.

ADPN, expressed in human and rat retinas, exerts protective effects in ocular pathologies, including diabetic retinopathy, retinopathy of prematurity, and age-related macular degeneration [33–35]. ADPN also accelerates healing in corneal alkali burns [36], reduces corneal neovascularization [37], and alleviates dry eye disease [38, 39]. While our study focuses on ADPN’s therapeutic potential in diabetic keratopathy, its expression in normal corneal epithelium may be modulated by environmental or physiological factors (e.g., circadian rhythms, tear film composition, basal metabolic demands). However, direct evidence for such regulation in the cornea remains scarce, representing a critical area for future investigation, particularly given ADPN’s protective roles in diabetic models.

To evaluate ADPN’s role in DK, we treated experimental models with ADPN. H-glu disrupted epithelial-neural-immune crosstalk, whereas ADPN restored this balance, providing further support for its therapeutic efficacy in ocular diseases, especially DK. ADPN exerts biological effects via binding to its receptors, AdipoR1 and AdipoR2. We confirmed expression of both receptors in murine corneas and observed downregulation of ADPN in H-glu-stressed cells. ADPN treatment increased insulin and p-AKT levels in diabetic mice (Supplementary Figure S7). To identify the mediating receptor, we used AdipoR1- and AdipoR2-specific siRNAs. Our results indicated that ADPN promotes corneal epithelial repair and nerve regeneration through AdipoR1 rather than AdipoR2, highlighting AdipoR1 as a key regulator of the “epineuroimmune” axis.

**Fig. 11:**
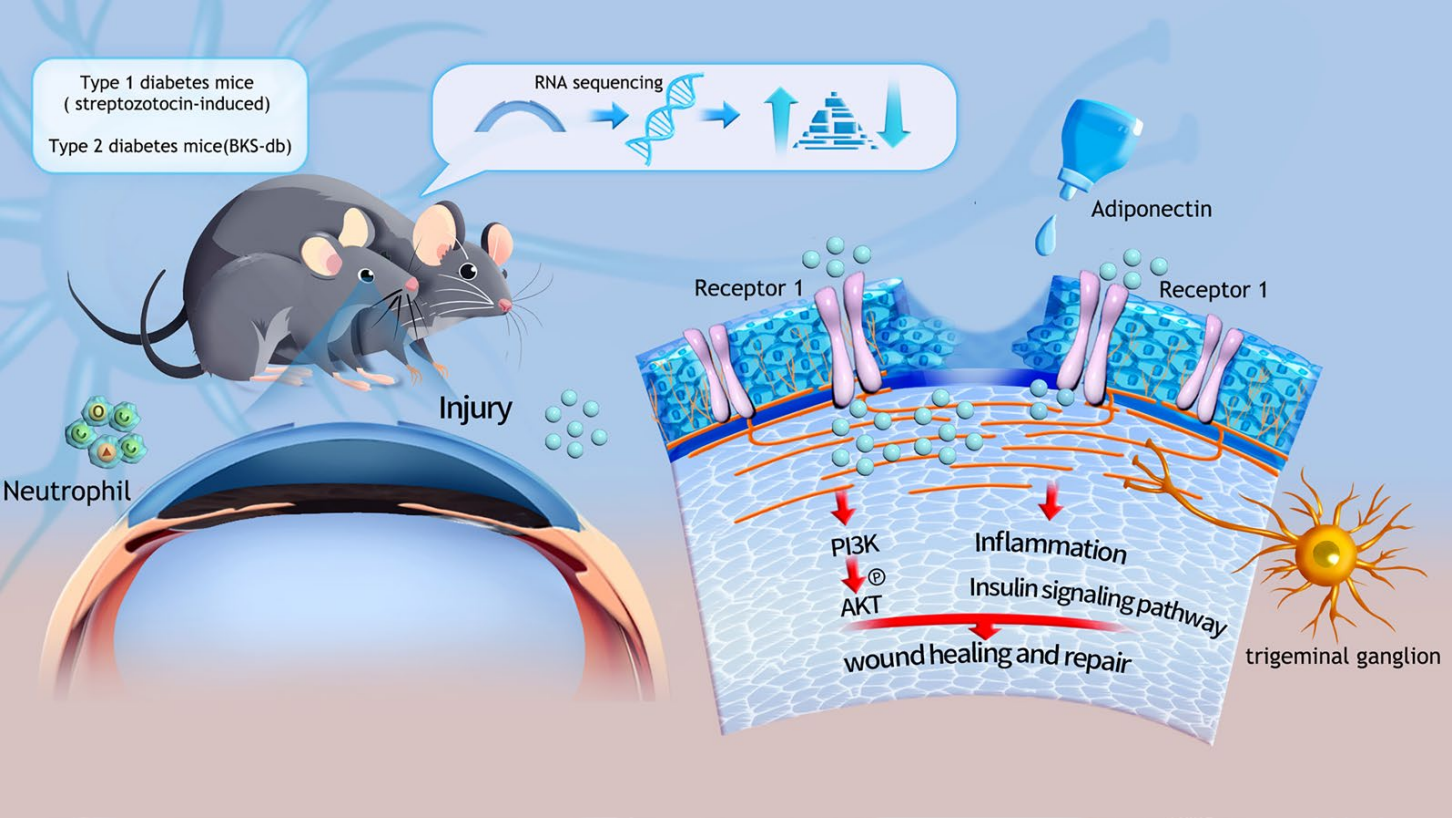
Schematic model of adiponectin (ADPN)-mediated corneal wound repair and nerve regeneration via the AdipoR1/AKT axis in diabetes. Locally applied ADPN binds to AdipoR1, activating AKT signaling to suppress neutrophil aggregation. Concomitantly, ADPN synergizes with insulin signaling to promote corneal epithelial and nerve repair, collectively accelerating wound closure

Impaired PI3K/AKT signaling is a hallmark of DK, contributing to delayed re-epithelialization [40]. Consistent with this, we observed reduced AKT activation in both type 1 and type 2 diabetes models; ADPN treatment restored AKT signaling. Disruption of AdipoR1 (but not AdipoR2) abolished ADPN-induced p-AKT activation, confirming AdipoR1’s role in mediating AKT pathway activation (Fig. 11).

Notwithstanding these findings, our study has limitations. First, the link between p-AKT signaling and other critical pathways (e.g., insulin signaling) remains unclear. Second, the biochemical dynamics of corneal nerve repair at the cellular level require further clarification. While prior studies have partially elucidated the role of insulin signaling in corneal nerve repair in type 1 diabetes [41], our preliminary data show that p-insulin receptor substrate 1 (p-IRS1) expression in diabetic corneal epithelia correlates with p-AKT levels; ADPN treatment upregulated both (Figures S7, S8). Given ADPN’s role in enhancing insulin sensitivity, its specific contribution to corneal nerve repair in diabetes warrants deeper investigation.

## Conclusions

In summary, H-glu impairs corneal epithelial wound healing and nerve repair while promoting neutrophil accumulation. ADPN regulates the “epineuroimmune” axis primarily through AdipoR1, with the AdipoR1/AKT pathway mediating its reparative effects. These findings suggest that ADPN holds promise as a therapeutic candidate for DK.

## Supplementary Information

Supplementary material 1.

## Data Availability

Not applicable.
