# Supplementary material for: The role of adiponectin and AdipoR1/AKT signaling axis in mediating diabetic corneal epithelial wound healing and sensory nerve regeneration
Source: Eye Vis (Lond). 2025 Oct 27;12:43. doi: 10.1186/s40662-025-00458-w (PMC12557978; doi:10.1186/s40662-025-00458-w)

**Supplementary Materials**

**Table S1.** Antibodies used for western blot analysis

| Antibody | Company | Catalogue No. | Dilution |
| --- | --- | --- | --- |
| m-AdipoR1 | Proteintech | 66619-1-Ig | 1:1000 |
| h-AdipoR1 | Invitrogen | SC69-04 | 1:1000 |
| m-AdipoR2 | Abcam | ab231051 | 1:1000 |
| h-AdipoR2 | Thermo Fisher | PA5-114166 | 1:1000 |
| Ki-67 | Abcam | ab16667 | 1:1000 |
| NF-κB | Cell Signaling Technology | 8242 | 1:1000 |
| p-AKT | Proteintech | 66444-1-Ig | 1:2000 |
| AKT | Proteintech | 10176-2-AP | 1:3000 |
| β-actin | Proteintech | 66009-1-Ig | 1:50000 |

**Figure S1. Adiponectin (ADPN) levels in sera, corneas, and trigeminal ganglia are reduced in type 2 diabetic mice compared with normal controls.​**​ ADPN concentrations were measured in serum, corneal tissues, and trigeminal ganglia from type 2 diabetic (db/db) mice and age-matched normal control mice. n = 6 per group. Data were analyzed using one-way ANOVA with Dunnett’s post-hoc test (for multiple groups) or unpaired two-tailed Student’s t-test (for two groups). **P* < 0.05 vs. normal control mice.

**
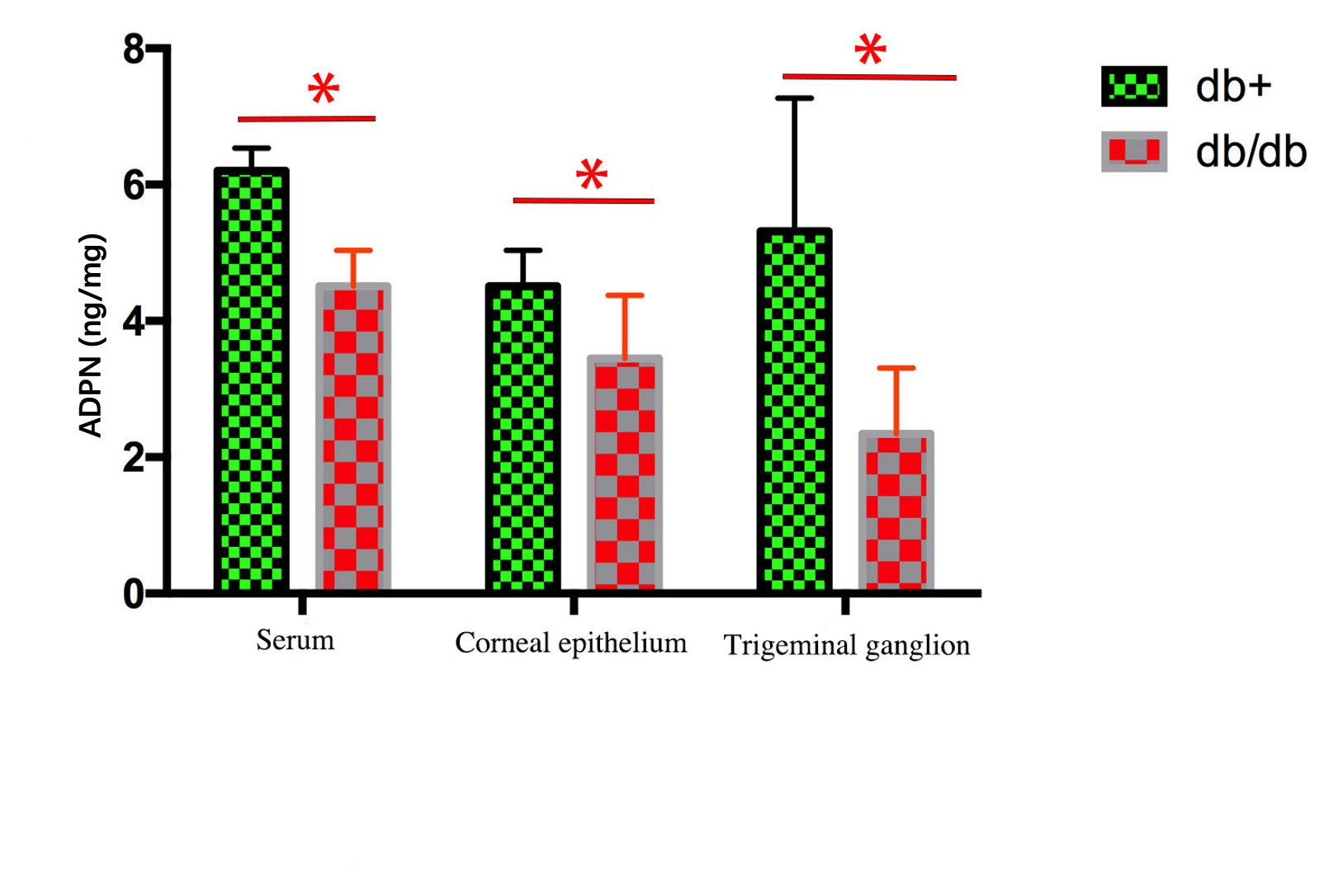
**

**Figure S2. Delayed corneal epithelial healing and nerve injury in diabetic mice. a** Corneal epithelial healing rate was significantly slower in diabetic mice compared with normal controls following a 3 mm diameter epithelial nerve injury. **b** The area ratio of corneal epithelial defects in diabetic mice was markedly larger than that in normal mice at 48 hours and 72 hours post-injury (***P* < 0.001 and **P* < 0.05, respectively). **c** Corneal nerve sensitivity in diabetic mice was reduced relative to normal mice 1 week after epithelial injury repair (***P* < 0.001). n = 6 mice per group. Data were analyzed using one-way ANOVA with Dunnett’s post-hoc test (for multiple groups) or unpaired two-tailed Student’s t-test (for two groups). **P* < 0.05, ***P* < 0.01 vs. normal control mice.

**
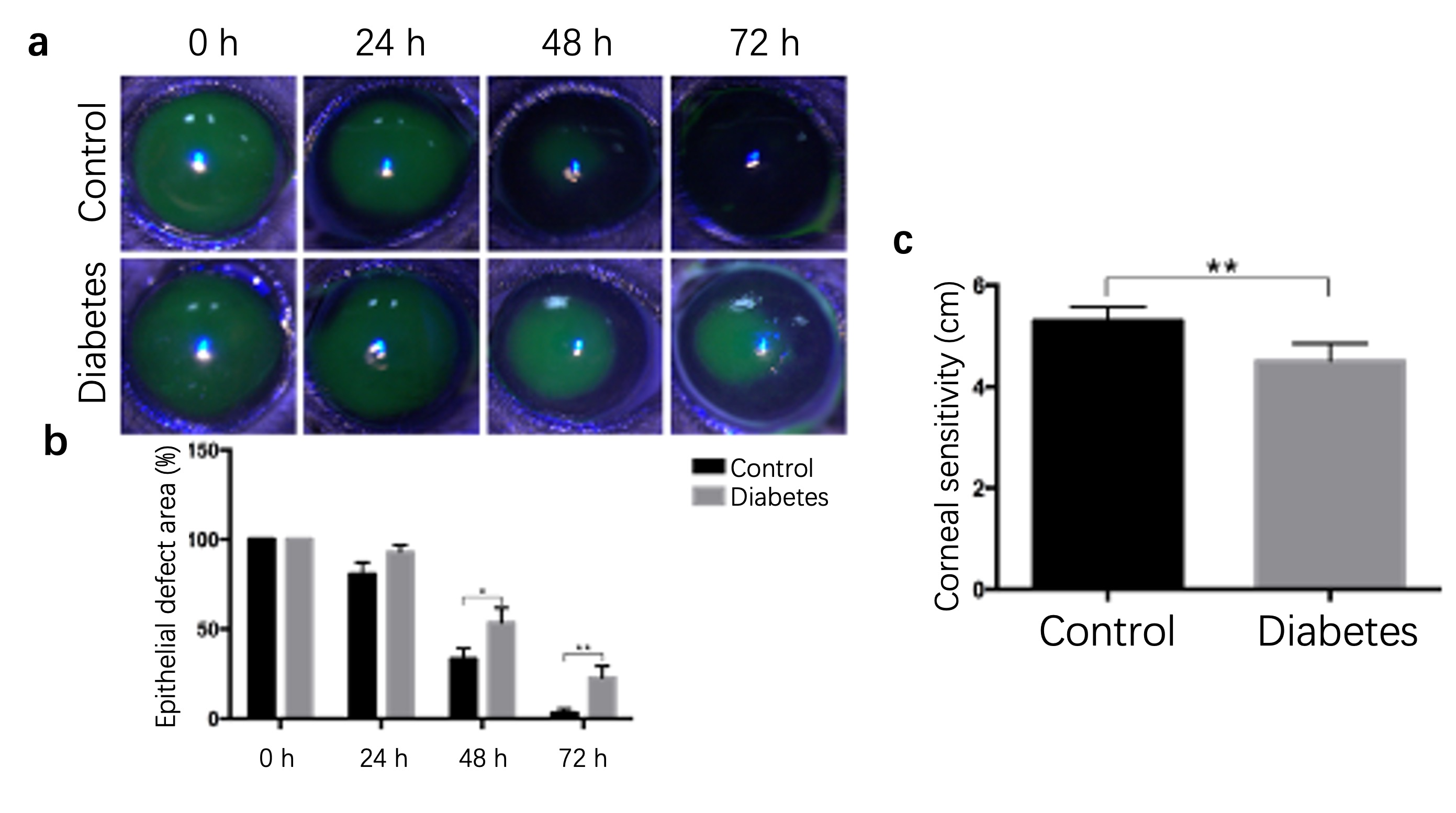
**

**Figure S3. Pathophysiological alterations in the cornea of type 1 diabetic mice.** This figure illustrates the structural and functional abnormalities in the cornea of streptozotocin (STZ)-induced type 1 diabetic mice compared with normal controls. **a** Fluorescein sodium staining revealed reduced integrity of the corneal epithelium in diabetic mice, characterized by irregular staining patterns and epithelial defects (indicated by arrowheads), whereas normal mice exhibited uniform and intact epithelial staining. **b** Tiger red staining highlighted increased degeneration or death of corneal epithelial cells in diabetic mice, manifesting as dense red-stained apoptotic/necrotic cells (arrowheads), in contrast to minimal staining in normal controls. **c** Histological examination (hematoxylin-eosin staining) demonstrated disorganized arrangement of corneal epithelial basal cells in diabetic mice, with loose and edematous basal layer structure, reduced nuclear density, and disrupted epithelial architecture (scale bar: 50 μm). **d** Terminal deoxynucleotidyl transferase dUTP nick end labeling (TUNEL) staining showed a marked increase in apoptotic cells (green fluorescence) in the corneal epithelium of diabetic mice, indicating elevated apoptosis (scale bar: 20 μm). **e** Corneal nerve sensitivity testing using a Cochet-Bonnet esthesiometer revealed significantly reduced sensory responsiveness in diabetic mice compared with normal controls (*P* < 0.001). n = 6 mice per group. Data were analyzed using one-way ANOVA with Dunnett’s post-hoc test (for multiple groups) or unpaired two-tailed Student’s t-test (for two groups). ***P* < 0.01 vs. normal control mice.

**
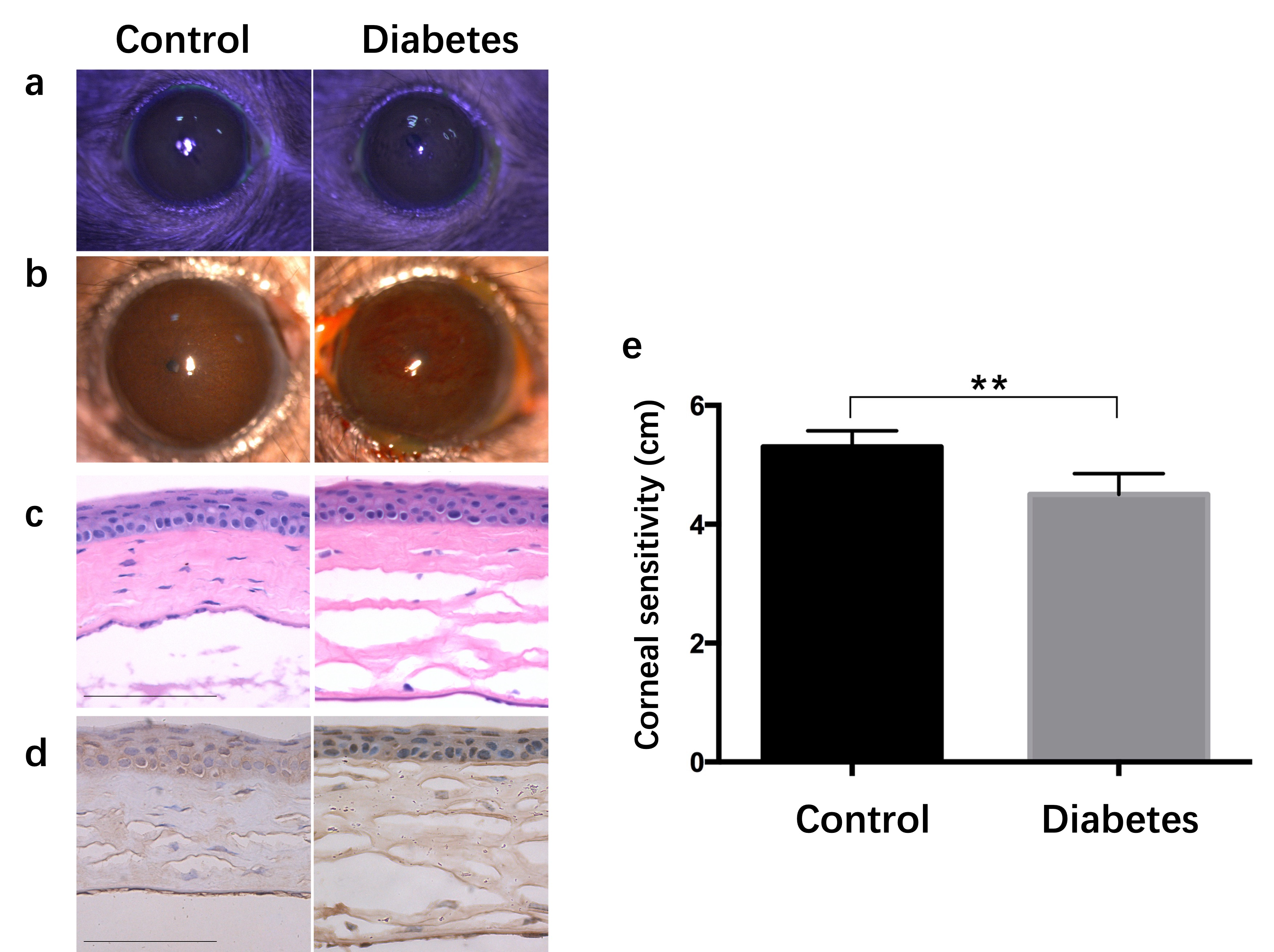
**

**Figure S4. Spatial distribution of AdipoR1 and AdipoR2 in the normal mouse cornea as detected by immunofluorescence staining. Representative images from three independent experiments are shown.**

**
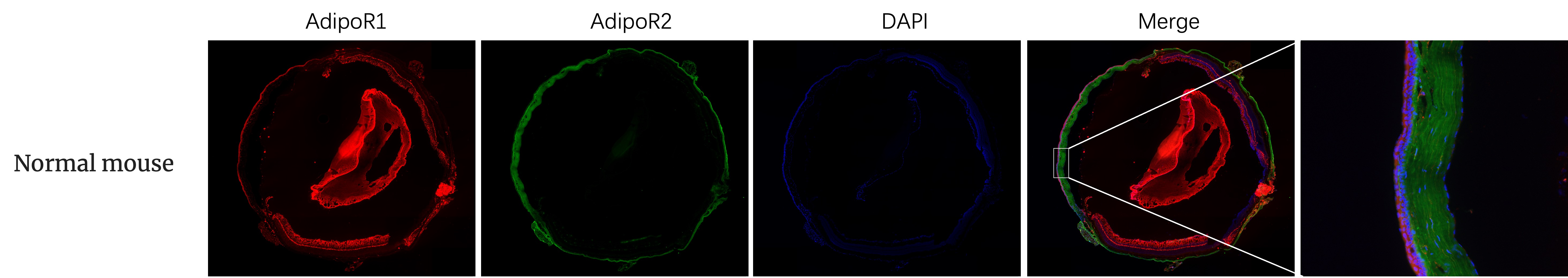
**

**Figure S5. Immunofluorescence staining reveals positive AdipoR1/R2 expression in normal human corneal epithelial cells (HCECs).** Representative immunofluorescence images of AdipoR1 and AdipoR2 staining in normal HCECs are shown. Positive staining (as indicated by fluorescence intensity) was detected in the cytoplasm and/or membrane of HCECs, consistent with the subcellular localization of adiponectin receptors. Images are representative of three independent cell culture experiments. Scale bar represents 25 μm.


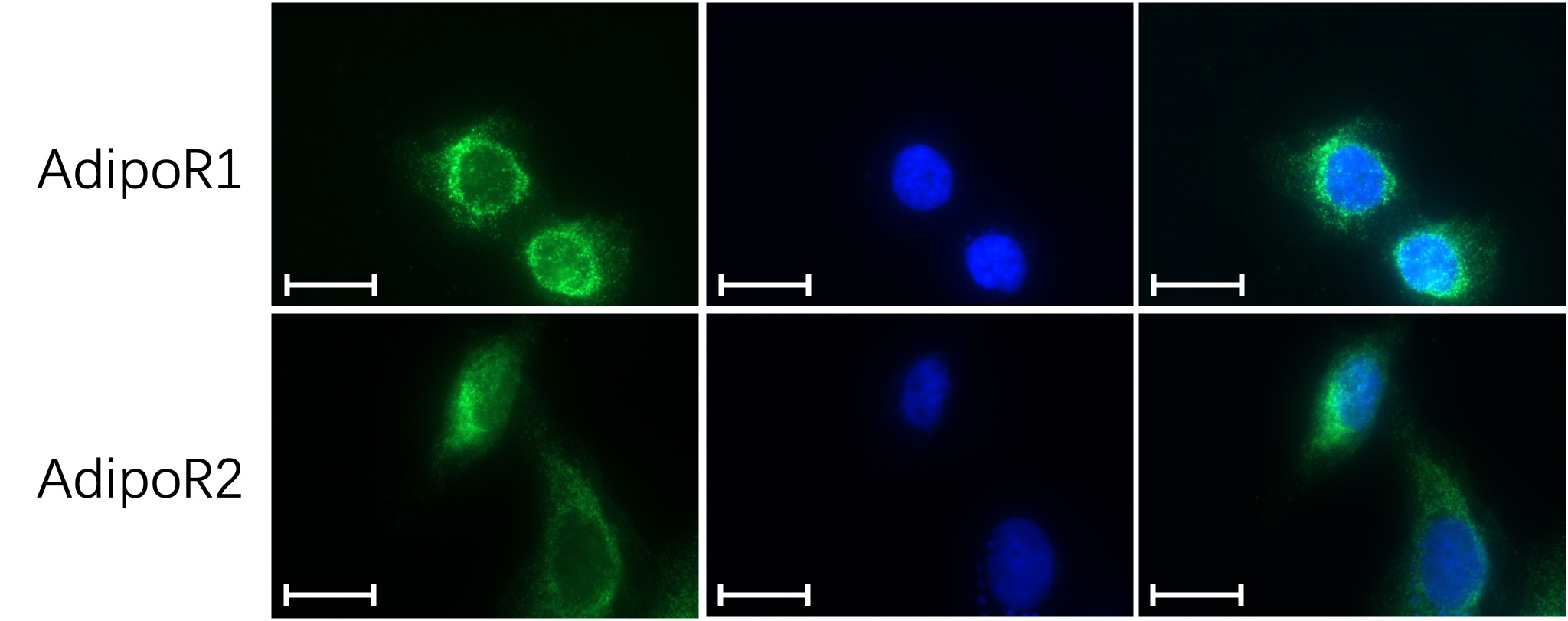


**Figure S6. Silencing of AdipoR1 or AdipoR2 expression using receptor-specific siRNAs and analysis of their spatial distribution in mouse corneas.** AdipoR1- or AdipoR2-specific siRNAs were transfected into mouse corneas to silence receptor expression. Immunofluorescence staining was performed to visualize the spatial distribution of AdipoR1 and AdipoR2, followed by semi-quantitative analysis of their expression levels. Representative immunofluorescence images from three independent experiments are shown.


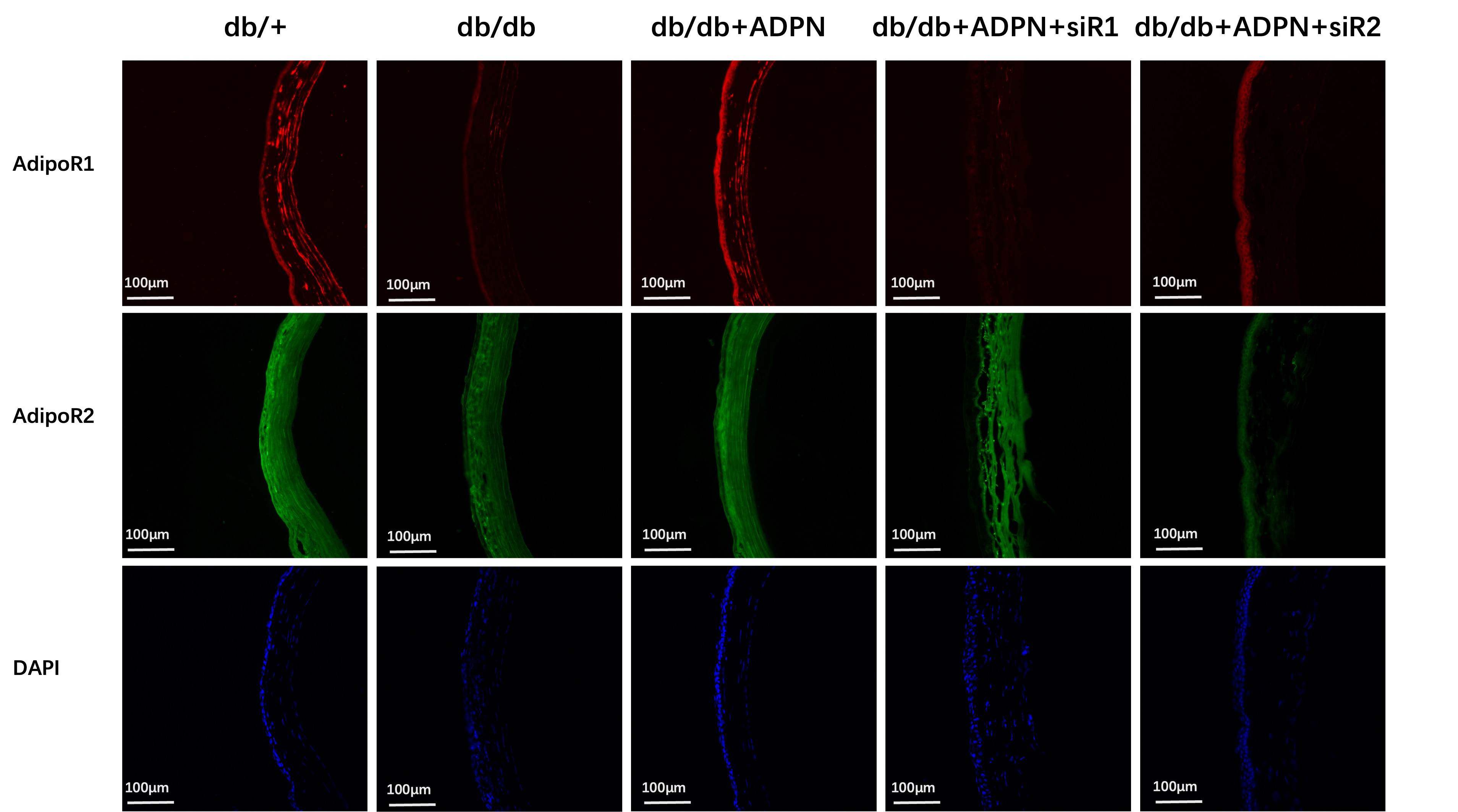


**Figure S7. Reduced activation of IRS1 and AKT signaling pathways during corneal injury healing in type 1 diabetic mice. a, c** Western blot analysis of phosphorylated insulin receptor substrate 1 (p-IRS1) and phosphorylated AKT (p-AKT) protein expression in diabetic mice during corneal epithelial injury healing. **b, d** Immunofluorescence staining of p-IRS1 (**b**) and p-AKT (**d**) in corneal epithelia of diabetic mice and normal controls. Fluorescence intensity of p-IRS1 and p-AKT was markedly weaker in diabetic mice compared with normal mice. Representative images from three independent experiments are shown. Reduced activation of IRS1 and AKT signaling correlates with impaired corneal epithelial repair in type 1 diabetes, consistent with the pathological phenotype of delayed wound healing.

**
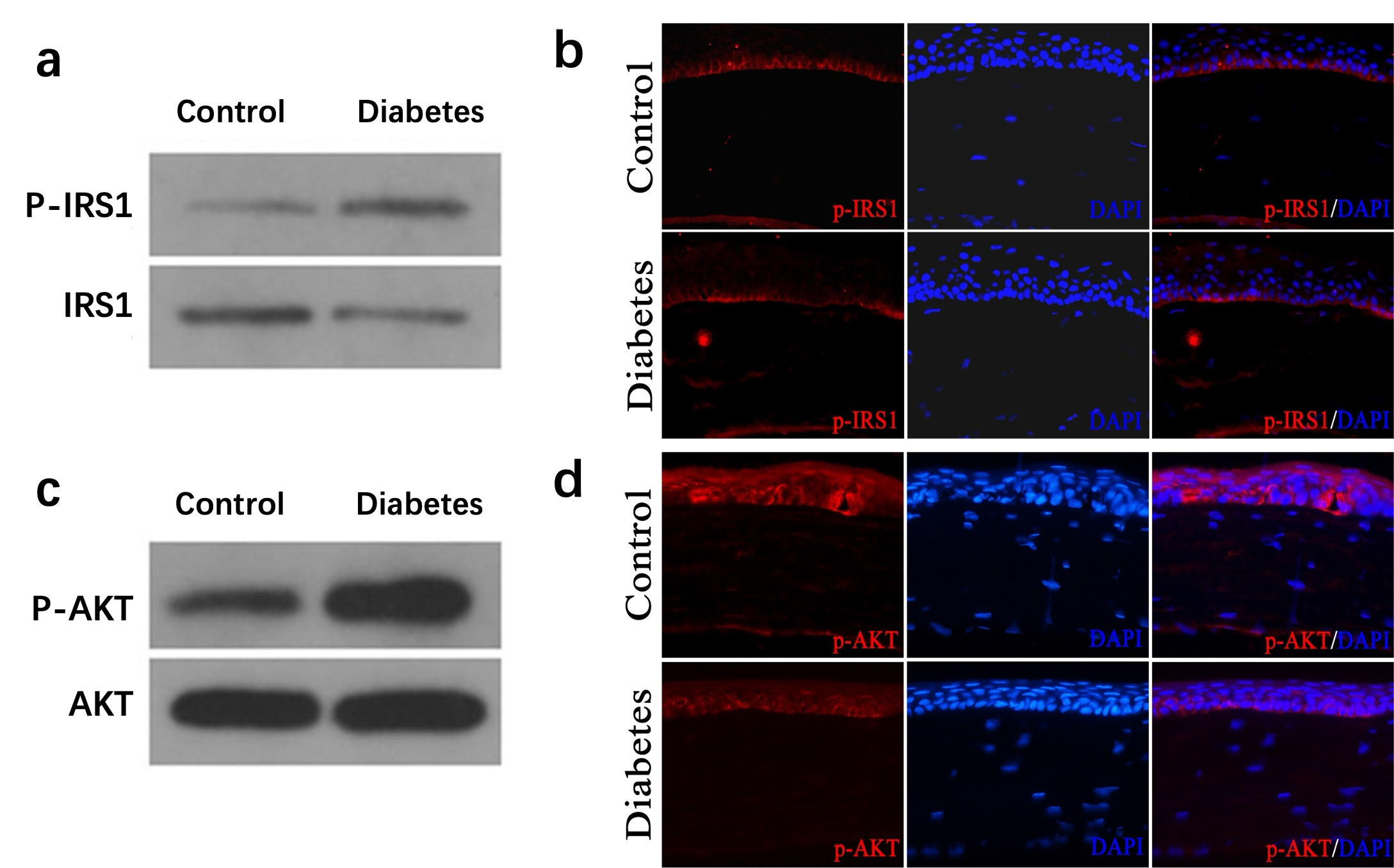
**

**Figure S8. Local application of adiponectin (ADPN) eye drops promotes activation of the p-IRS1 and p-AKT signaling pathways in the corneas of type 1 and type 2 diabetic mice.**


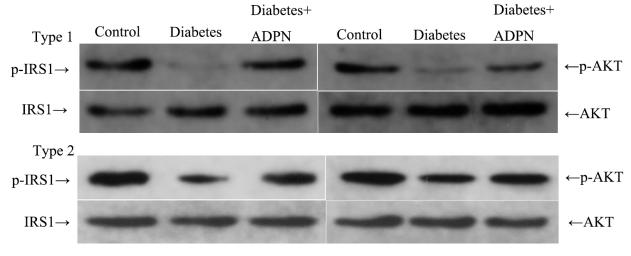

Supplement: Supplementary file 1 — Supplementary material 1. [file 40662_2025_458_MOESM1_ESM.docx]
